# Comparative analysis of venom genes in the chromosome-level genomes of two closely related cone snails

**DOI:** 10.1186/s12864-026-12745-4

**Published:** 2026-03-18

**Authors:** Ana Herráez-Pérez, Rafael Zardoya

**Affiliations:** 1 Department of Biodiversity and Evolutionary Biology, Museo Nacional de Ciencias Naturales (MNCN-CSIC), José Gutiérrez Abascal 2, Madrid, 28006 Spain; 2 Doctoral School, Universidad Autónoma de Madrid (UAM), Francisco Tomás y Valiente 2, Madrid, 28049 Spain

**Keywords:** Cone snails, Venomics, Transcriptomics, Comparative genomics, Synteny, Venom gene families

## Abstract

**Background:**

Venom cocktails of cone snails are characterized by their dynamic composition and the rapid evolution of toxin-encoding genes due to various selective pressures. The integration of comparative genomics, transcriptomics, and proteomics has emerged as a crucial approach for the comprehensive characterization of venom repertoires and the understanding of venom gene evolution.

**Results:**

The venom gland transcriptomes of three individuals of the Canary Island cone snail *Kalloconus canariensis* had 586 different transcripts, and showed remarkably high intraspecific variability. These transcripts were used to annotate and locate venom genes in the assembled pseudochromosomes of the *K. canariensis* genome, and comparative genomic analyses were performed with the venom genes of the closely related Mediterranean cone snail *Lautoconus ventricosus*. A total of 86 orthogroups with counterparts in both genomes were identified, comprising 118 and 159 venom genes from *K. canariensis* and *L. ventricosus*, respectively. Synteny analyses comparing the two genomes revealed that most (86%) venom genes were located in relatively conserved genomic regions within homologous pseudochromosomes. Notwithstanding, up to 16 genes were rearranged together with their flanking regions into non-homologous pseudochromosomes, suggesting local genomic reorganizations and/ or transpositions, likely mediated by transposable elements.

**Conclusions:**

Comparative analyses of *K. canariensis* and *L. ventricosus* genomes revealed a core set of venom genes common to both species, showing overall synteny conservation. In addition, numerous instances of lineage-specific venom locus configurations were detected, suggesting that local tandem gene duplications and losses are major drivers of diversification for this gene family.

**Supplementary Information:**

The online version contains supplementary material available at 10.1186/s12864-026-12745-4.

## Background

Biological diversity arises from the complex interaction of multiple evolutionary forces acting on genomic variation. Mechanisms such as mutation, genetic drift, recombination, gene duplication, and natural selection, all contribute to generating and shaping genetic diversity within and between species [1, 2]. Among these mechanisms, gene duplication is a significant source of evolutionary novelty, with duplicated genes retained through processes such as neofunctionalization, subfunctionalization, or dosage amplification [3–5]. Yet, the relative contribution of these processes, as well as the mechanisms driving the expansion and contraction of gene families, remain challenging to disentangle [6, 7]. Within animal lineages, venomous species offer an outstanding system to study these dynamics, as venom composition is shaped by the rapid evolution and turnover of complex multigene families.

Animal venoms are complex cocktails, primarily made of proteins and peptides, including enzymes (like proteases, phospholipases, nucleases) and non-enzymatic toxins (like neurotoxins, cytotoxins, cardiotoxins, etc.), alongside smaller amounts of other molecules like lipids, metal ions, and carbohydrates [8, 9]. The advent of high-throughput technologies that produce massive genomic, transcriptomic, and proteomic data, has revolutionized venom research [10–14]. Venomics integrates the above mentioned three types of data to identify and catalogue the diverse venom components, and thus, is greatly accelerating the discovery of novel drugs for pharmacology and human therapeutics [15, 16].

Cone snails (Caenogastropoda: Conidae) have evolved one of the most sophisticated venom systems among marine invertebrates. With more than 900 described species, cone snails represent a sound model system to study the origin and diversification of venoms [17, 18]. Each cone species produces a complex mixture of hundreds of small (10–30 amino acids), cysteine-rich peptides known as conotoxins, which mainly target ion channels and neuromuscular receptors, triggering diverse physiological responses in prey, including sedation, tetanic paralysis, or even death [19].

The venom cocktails of cone snails are being catalogued actively through transcriptomics and proteomics of the venom gland [12, 20–23]. In contrast, the generation of high-quality chromosome-level genome assemblies of cone snails has lagged behind until recently. Only two chromosome-scale genomes of cone snails have been published to date: that of the Mediterranean cone snail, *Lautoconus ventricosus* [13] and that of the endemic Canary Island cone snail, *Kalloconus canariensis* [24]. The genome assembly of the Chinese tubular cone snail, *Dendroconus betulinus* was reported as chromosome level [25] but the scaffolded assembly is not available in the National Center for Biotechnology Information (NCBI) Bioproject: PRJNA665547. Finally, the genome of the “cloth-of-gold” cone, *Cylinder textile* has been recently reported [26], although the sequence data for this genome is not yet publicly available.

As is the case in other multigene families, the extraordinary diversity of conotoxins is thought to arise from the combination of multiple evolutionary mechanisms, including gene duplication, (ectopic) recombination, accelerated substitution rates, exon shuffling, alternative splicing, differential expression, and post-translational modifications, among others [26–31]. In this regard, the addition of comparative genomic studies to the already growing research on comparative transcriptomics and proteomics (e.g. [12, 23, 32–34]), is essential to discern among competing hypotheses on the origin and diversification of conotoxins and other venom-related proteins.

In this study, we aim to contribute to the understanding of the evolutionary mechanisms underpinning the great diversification of cone snail venom proteins by comparing the genomes of *L. ventricosus* [13] and *K. canariensis* [24]. The specific objectives of this study are: (i) to annotate and locate venom genes in the *K. canariensis* genome using the venom gland transcriptome; (ii) to identify those venom genes that are common to both species, and thus, likely essential for a generalized venom function; and (iii) to determine potential translocation, duplication and/or loss events in the venom gene families. Altogether, the comparative analyses performed in this study should provide novel insights on the major genomic mechanisms driving the diversification of the multigene families involved in the generation of the great variety of lineage-specific complex venom repertoires of cone snails.

## Materials and methods

### RNA extraction, library preparation, sequencing and transcriptome assembly

Adult specimens of *K. canariensis* were collected in Tenerife, Canary Islands, in 2020 [24]. The venom glands of two of them (TF42 and TF43) were previously RNA sequenced for genome annotation [24]. For the present study, the RNA of the venom gland of a third specimen (TF39) was sequenced (in order to achieve a minimum number of independent biological replicates [35]).

Briefly, the venom gland tissue of TF39 was extracted with TRIzol LS reagent (Thermo Fisher Scientific, Waltham, MA, USA). The Direct-zol RNA miniprep kit (Zymo Research, Irvine, CA, USA) was used to purify 25 µg of total RNA, following manufacturer’s instructions. Library construction and sequencing was performed at NIMgenetics (Madrid, Spain). Paired-end sequencing (2 × 100 bp) was performed in an Illumina NovaSeq 6000 platform, which produced 220 million raw paired-end reads with a read quality (Q30) above 85%.

The transcriptomes of the three samples were assembled both *de novo* and using the genome as reference. Briefly, raw Illumina reads were quality checked using FastQC v0.11.9 [36]. In Trinity v2.15.0 [37], reads were filtered and trimmed using the trimmomatic option (that removed Illumina adapters, trimmed low-quality regions and discarded short sequences; SLIDINGWINDOW: 4; LEADING: 5; TRAILING: 5; MINLEN: 25), and *de novo* assembled with default parameters (minimum contig length: 200; sequence identity threshold: 0.95). For genome-guided assembly, the trimmed Illumina paired-end reads were aligned to the genome assembly using minimap2 v2.30-r1287 [38] in spliced alignment mode. Alignments were sorted and merged with samtools v1.23 [39], and the resulting BAM file was used as input for genome-guided assembly with Trinity v2.15.0 [37], specifying a maximum intron length of 30 kb. Completeness of the transcriptome assemblies was checked using BUSCO v5.1.3 [40] with the metazoan_odb10 set.

### Annotation of the venom transcriptome

A custom reference amino acid database was generated with all conotoxin precursors, hormones, and associated venom proteins of cone snails available in the NCBI non-redundant database [41], Uniprot [42], and ConoServer [43]. Redundancy from merged databases was eliminated using CD-HIT v4.8.1 [44] with a 95% amino acid identity threshold. Transcripts were annotated using BLASTX similarity searches against the above custom reference database (e-value of 1.00E-05). TBLASTX similarity searches against the NCBI non-redundant database, along with a thorough manual curation, were also performed in order to discard false-positive hits (not corresponding to canonical conotoxins) or assembly artifacts (in low-coverage terminal positions and chimeras). Highly truncated (missing > 55% of the estimated total length) peptide sequences were removed to produce the final working list of conotoxin precursors, hormones, and venom-related proteins of. *K. canariensis*.

The Conoprec tool [43] was used to identify the three domains (signal, propeptide, and mature) of predicted conotoxin precursors and the cysteine frameworks of the mature functional peptides. Conotoxin precursors were assigned to different protein superfamilies based on the two highest scoring full-length hits (with a percentage of sequence identity > 70%) in the BLASTP searches, using the relatively conserved signal domain as query.

### Mapping of conotoxin genes in the genome

The whole-genome sequence and annotation files of *K. canariensis* (BioProject PRJNA843968; [24] were downloaded from NCBI. In parallel, a custom transcriptome-based reference database was constructed compiling venom genes from *K. canariensis* (this study) and those from related cones: *Chelyconus ermineus* [20], *Pionoconus magus* [22], several *Africonus*,* Varioconus*, and *Kalloconus* species [21], *L. ventricosus* [13], and two *Virroconus* species [23].

BITACORA v1.4 [45] was used in full mode to perform similarity-based searches with BLAST v2.13.0+ [46] and Hidden Markov Model (HMM)-based searches with HMMER v3.4 [47, 48] in the genome against the reference database, using two different e-value cutoffs (1.00E-05 and 1.00E-03). The resulting output was loaded into Geneious Prime v2024.0.2 [49], together with the genome annotation. Each predicted gene was (1) manually curated by comparing exons with the predicted venom gene transcripts and adjusting intron-exon GT/AG boundaries to detect any broken open reading frame and possible missing exons, as well as (2) located in the different pseudochromosomes. Finally, transcript isoforms were assigned to the corresponding venom gene through BLASTP searches.

### Comparative analysis of venom gene regions

Venom genes of both species were classified into orthogroups (OGs) [50] using Orthofinder v2.5.5 [51], with default parameters. To do so, the amino acid sequences of the reference database used as input for BITACORA (see above) plus the annotated venom genes of the *K. canariensis* and *L. ventricosus* genomes were used to conduct similarity searches and reconstruct phylogenetic trees.

To determine whether venom genes from the same OG were located within conserved flanking regions or not, a fragment spanning the 200 kb upstream and downstream regions of each annotated venom gene was delimited using Satsuma2 [52] in the *K. canariensis* and *L. ventricosus* genomes, and aligned using pairwise BLASTN. The percentages of sequence identity and alignment coverage of the pairwise alignments were calculated to quantify the level of conservation of flanking regions between species. Additionally, this comparative framework enabled distinguishing whether those OGs that are located in non-homologous pseudochromosomes translocate alone or accompanied by their flanking regions.

## Results

### Diversity of venom gland transcripts in *K. canariensis* compared to *L. ventricosus*

The Illumina RNA-seq of the venom gland libraries of three specimens of *K. canariensis* (TF39, TF42, TF43; Supplementary Table S1) produced an average of 31,529,653 paired reads, and the number of assembled transcripts varied between 212,273 and 707,446 (mean of 460,171). BUSCO recovered completeness scores of 60%, 78%, and 53% for TF39, TF42, and TF43 transcriptomes, respectively.

A total of 586 different transcripts encoding conotoxin precursors, hormones and venom-related proteins involved in the processing of conotoxins or enhancing venom activity were identified and annotated in the three analyzed venom gland transcriptomes. Of these transcripts, 499 were classified into 56 conotoxin precursor superfamilies on the basis of the divergence of the signal domain and the presence of different cysteine frameworks; 16 were assigned to nine hormone gene families; and 71 were classified into 21 gene families encoding various proteins related to venom synthesis or function (Supplementary Table S2; Supplementary Files S1 and S2).

**Fig. 1:**
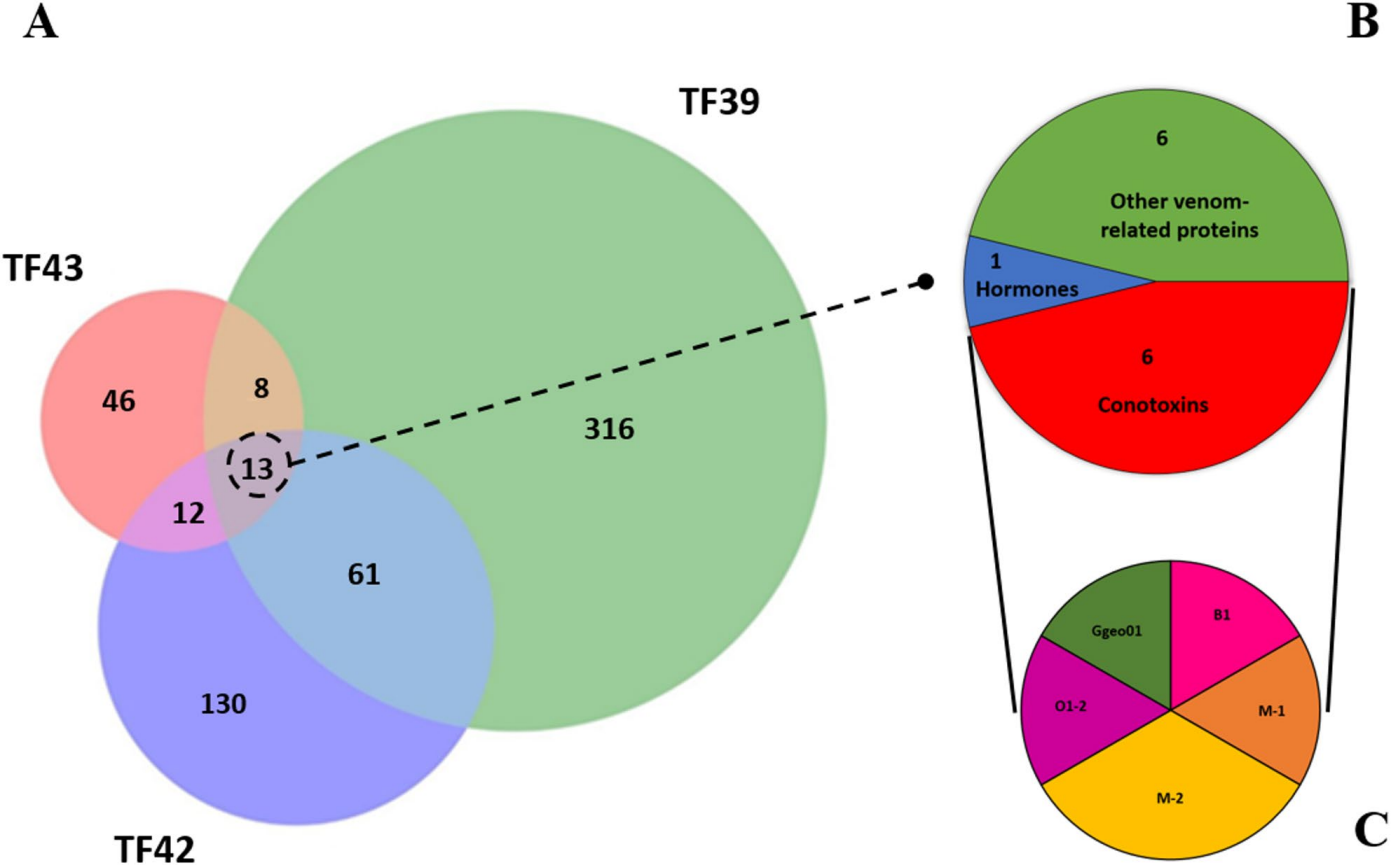
Venn diagram representation of *K. canariensis* transcriptomes. (**A**) Venn diagram representation of transcripts that were shared or unique to the venom glands of the three *K. canariensis* individuals (TF36, 39, and 42). Circle areas are proportional to the number of venom transcripts. For the shared sequences, the proportions of the different conotoxin precursor, hormone and venom-related sequences (**B**) and conotoxin precursor superfamilies (**C**) are shown

Significant differences in relative quantity and diversity of transcripts were observed between the three venom gland transcriptomes. A total of 13 transcripts were identical in the three individuals, including six conotoxin precursors, one hormone, and six venom-related proteins. The shared conotoxin precursors comprised one member each of B1, M1, O1, and Ggeo01 superfamilies, and two members of the M2 superfamily (Fig. 1). Up to 74 transcripts were common to TF39 and TF42, 21 were shared between TF39 and TF43, and 25 between TF42 and TF43 (Fig. 1; Supplementary Table S2; Supplementary File S1).

The venom gland transcriptomes of the individuals used for the annotation of the genome assemblies of *K. canariensis* (TF39) and *L. ventricosus* (CV8) [13] were compared in order to assess differences in the composition of conotoxin precursor superfamilies between the two species. A total of 398 transcripts were found in *K. canariensis* (374 conotoxin precursors, 6 hormones, and 18 other venom-related proteins) whereas 289 were identified in *L. ventricosus* (245 conotoxin precursors, 11 hormones, and 33 other venom-related proteins). M, O1, O2, and T superfamilies were the most diverse for both species (Supplementary Table S2).

### Venom gene diversity and distribution in the genome of *K. canariensis* compared to *L. ventricosus*

**Fig. 2:**
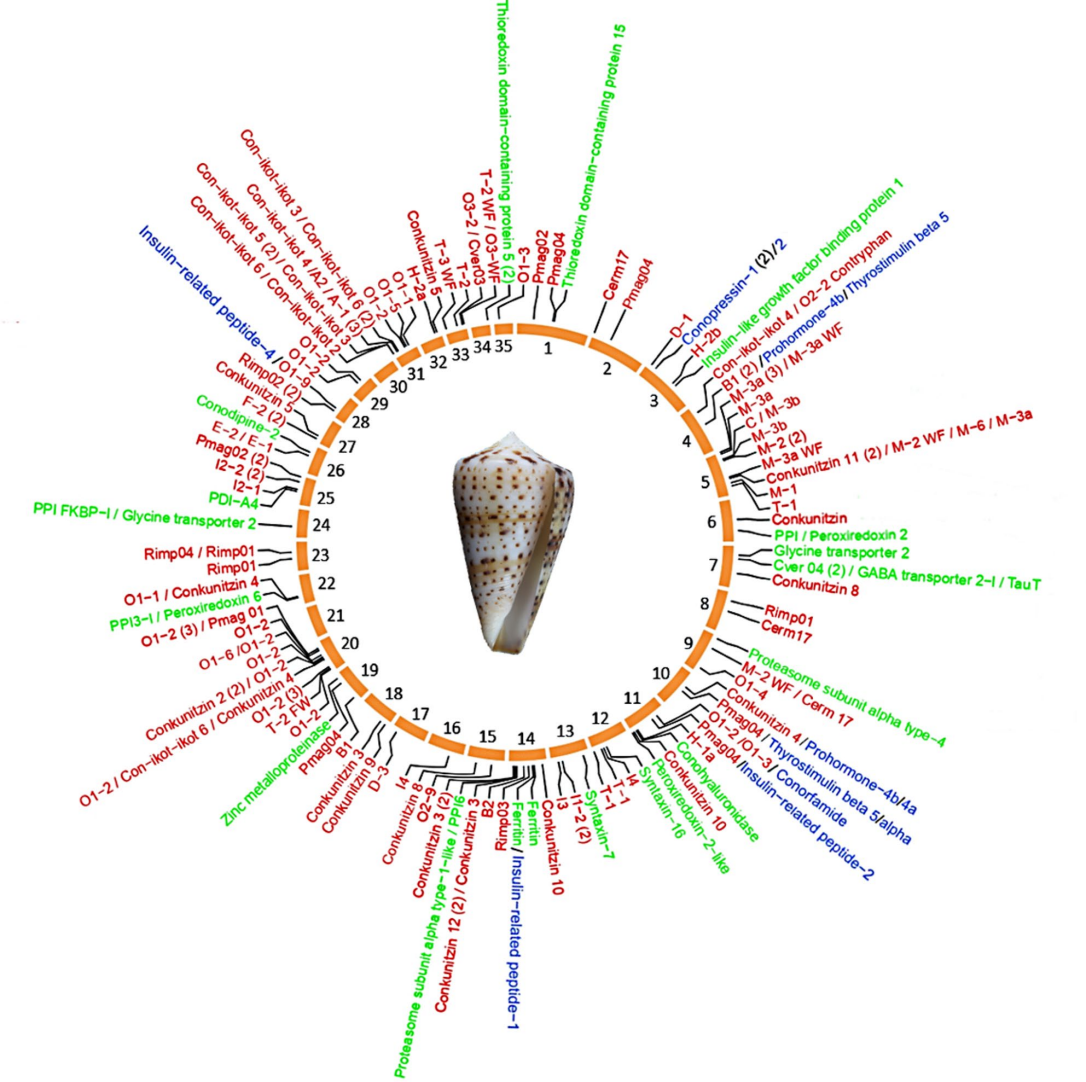
Distribution of venom genes in *K. canariensis* genome. Venom genes mapped into the *K. canariensis* genome. The distribution of the conotoxin precursor (red), hormome (blue), and venom-related (green) genes in the 35 pseudochromosomes is shown. Genes located closer than 2Mb were clustered and their number annotated in parentheses. A picture of the TF39 individual (voucher accession number MNCN15.05/94850) is shown

Venom transcripts of the three individuals of *K. canariensis* were used to annotate and locate the corresponding genes across the assembled pseudochromosomes in the genome of this species (Fig. 2; Supplementary Files S3, S4, and S5). Genome searches using a 1.00E-05 e-value cutoff recovered conotoxin precursor and venom-related genes but missed hormone genes, which were recovered using a 1.00E-03 e-value cutoff. The total number of recovered conotoxin precursor and venom-related genes was the same regardless of the cutoff value (see below). A total of 167 genes were located complete in the 35 pseudochromosomes, two additional genes were partially located in pseudochromosomes and completed manually with hits located in smaller scaffolds, and one extra gene was found entirely within a smaller scaffold (Fig. 2; Supplementary Table S3; Supplementary File S6). In comparison, in *L. ventricosus* 154 genes were located complete in the 35 pseudochromosomes and 79 were completed manually from smaller scaffolds, contigs, and raw reads [13]. In order to assign transcripts (query) to genes (target) in *K. canariensis*, we performed BLASTP similarity searches with different e-value cutoffs. Of the 586 transcripts detected in the three venom gland transcriptomes, 463 (79%), 474 (81%), and 489 (83%) transcripts were assigned to 119–120 out of the 170 genes using 1.00E-05, 1.00E-04, and 1.00E-03 e-values, respectively. For the 1.00E-04 cutoff, up to 33, 16 and 20% of the genes correlated with one, two, or more transcripts, respectively.

Of the 169 venom genes detected in the *K. canariensis* genome, 130 corresponded to conotoxin precursors, 14 to hormones, and 25 to venom-related proteins. The most abundant superfamilies in both *K. canariensis* and *L. ventricosus* were O1 (28 genes vs. 80), conkunitzin (21 vs. 26), M (14 vs. 21), and I (9 vs. 15), indicating a core set of expanded superfamilies (Supplementary Tables S3 and S4). Interestingly, the *K. canariensis* genome showed an expansion of the Con-ikot-ikot (11 genes vs. 3 in *L. ventricosus*) and Pmag (9 genes vs. 2 in *L. ventricosus*) superfamilies, whereas *L. ventricosus* exhibited broader representation of O1, T, Cerm and multiple minor superfamilies (e.g., J, K, L, N, P, Q, S, U, V, Y, Ggeo, Rmil; the latter being absent in *K. canariensis*, see Supplementary Table S4).

**Fig. 3:**
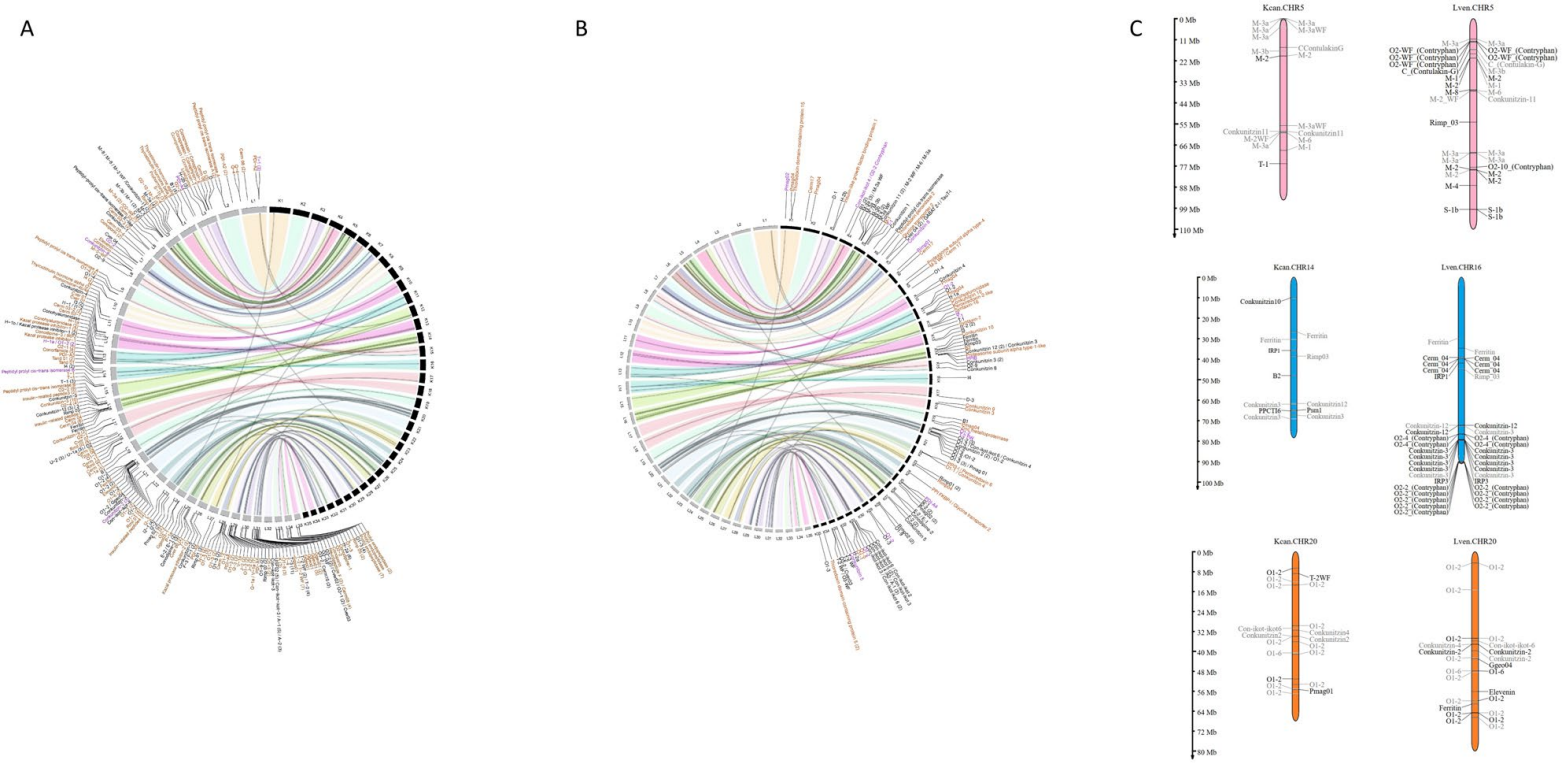
Synteny plot of *K. canariensis versus L. ventricosus*. Conserved synteny plot of the 35 pseudochromosomes between *K. canariensis *(right, black; K1 – K35) and *L. ventricosus* (left, grey; L1 – L35). Black lines (links) correspond to venom genes shared between both species. Labels indicate all the venom genes of *L. ventricosus* (**A**) and *K. canariensis* (**B**). Black labels are venom genes located in homologous pseudochromosomes; purple labels are venom genes located in non-homologous pseudochromosomes; and orange labels correspond to non-shared venom genes between the two species. (**C**) Pairwise comparisons between homologous pseudochromosomes particularly rich in venom genes show differences in genomic distribution of these genes between K. canariensis and L. ventricosus

Although venom genes were scattered across the genome of *K. canariensis* (Fig. 2), their distribution did not correlate with pseudochromosome size (R² =6 × 10^− 10^; *P* = 0.99; Supplementary Fig. S1), similarly to what was observed in *L. ventricosus* [13]. In *K. canariensis*, pseudochromosomes 5, 14 (homologous to pseudochromosome 16 in *L. ventricosus*), 20, and 31 (30 in *L. ventricosus*) were particularly rich in conotoxin precursor genes (Fig. 3; Supplementary Fig. S2), whereas pseudochromosomes 16 (14 in *L. ventricosus*), 17 (18 in *L. ventricosus*), and 35 (34 in *L. ventricosus*) barely had one or two venom genes. Only pseudochromosomes 21, 30, and 34 lacked any of these genes completely. This pattern is consistent with *L. ventricosus* for the last two pseudochromosomes (homologous 31 and 35, respectively) that likewise did not contain any conotoxin precursor gene [13] (Fig. 3). In *K. canariensis*, regions of local enrichment consistent with potential gene arrays were detected for the O1 superfamily on pseudochromosome 20 and for the conkunitzin superfamily on pseudochromosome 14, partially overlapping with those described in *L. ventricosus*. Similarly, in *L. ventricosus*, putative arrays of venom-related genes were reported in pseudochromosomes 4 (B1 superfamily), 16 (conkunitzin), 20 and 28 (O1), and 23 (I2) [13].

Genes encoding hormones were located in pseudochromosomes 3, 4, 10, 11, 14, and 28 of *K. canariensis* (Fig. 2; Supplementary Table S3). In both *K. canariensis* and *L. ventricosus*, homologous pseudochromosomes 3 harbored conopressin/conophysin 1 and 2 genes. Similarly, pseudochromosomes 4 and 10 in *K. canariensis* (homologous to 4 and 11 in *L. ventricosus*, respectively) contained both prohormone 4 (a and b) and thyrostimulin (α and β) genes. Pseudochromosome 11 of *K. canariensis* had one conorfamide gene and the homologous pseudchromosome 13 in *L. ventricosus* had five conorfamide genes. Finally, one copy of the insulin-related peptide gene was found each on pseudochromosomes 14 and 28 of *K. canariensis*, whereas one and four copies were found on the non-homologous pseudochromosome 13 and 21 of *L. ventricosus*, respectively.

Genes encoding venom-related proteins were found in pseudochromosomes 1, 3, 6, 7, 9, 11–14, 19, 22, 24–26, and 34 (Fig. 2; Supplementary Table S2). We annotated a total of 25 venom-related genes in the *K. canariensis* genome, compared to the 52 genes reported in the *L. ventricosus* genome [13]. The most common venom-related genes encoded proteasome subunits (3 genes located in pseudochromosomes 4, 9, and 14), peptydil-prolyl cis-trans isomerases (4 genes in pseudochromosomes 6, 14, 22, and 24), thioredoxin domain-containing proteins (4 genes in pseudochromosomes 1, 6, and 34), and syntaxins (3 genes in pseudochromosomes 12 and 13). In *K. canariensis*, pseudochromosomes 7 and 14 were particularly enriched in venom-related genes. In comparison, the *L. ventricosus* genome exhibited larger arrays and higher copy numbers of several families, including protein disulfide isomerase (PDI) paralogs (8 genes in pseudochromosomes 1–3, 14, 26, 28 and 29), Kazal-type protease inhibitors (7 genes in pseudochromosomes 9, 13, and 25), and prolyl endopeptidases (10 genes in pseudochromosome 35).

Furthermore, several gene families were species-specific, and for example, conoporin, elevenin, Kazal-type protease inhibitor, and prolyl endopeptidase, and neuropeptide F were found exclusively in *L. ventricosus*, whereas two membrane transporters (Glycine transporter 2 and Taurine transporter-like protein) and multiple thioredoxin-domain proteins were only detected in *K. canariensis*. Other gene families, such as conohyaluronidases and conodipines, were present in both species but with different copy numbers and chromosomal distribution, consistent with lineage-specific gene expansions or losses.

### Venom gene orthogroups in *K. canariensis* and *L. ventricosus* genomes

To investigate evolutionary relationships of venom genes between *K. canariensis* and *L. ventricosus*, we identified OGs using phylogenetic reconstructions. Of the 169 venom genes in *K. canariensis* and the 233 in *L. ventricosus*, a total of 86 OGs with counterparts in both genomes were identified, comprising 118 and 159 venom genes from *K. canariensis* and *L. ventricosus*, respectively (Supplementary Table S5). Within these OGs, 40 had only one copy in each species and thus, were considered orthologous. The remaining OGs had gene duplications rendering up to a maximum of seven and eight paralogs in *K. canariensis* and *L. ventricosus*, respectively (Supplementary Table S5). Additionally, 23 OGs included venom genes from *K. canariensis* and representatives from other cone species but *L. ventricosus*. Similarly, 123 OGs included venom genes from *L. ventricosus* and representatives from other cone species but *K.* c*anariensis* (Supplementary Table S5).

Of the 40 orthologous single-copy venom gene pairs, 38 were located within homologous pseudochromosomes, whereas two were found in non-homologous pseudochromosomes between the two species (Supplemnetary Table S5; Supplementary Fig. S2). Of the 78 venom genes of *K. canariensis* included in the 46 OGs containing paralogous genes, 64 and 14 genes were found in homologous and non-homologous pseudochromosomes, respectively (Supplementary Table S5; Supplementary Fig. S2).

To further understand the dynamics of venom gene evolution and diversification, we compared the non-coding genomic regions flanking venom genes between *K. canariensis* and *L. ventricosus* (Supplementary Table S6). In the 86 OGs with counterparts in both genomes, all flanking regions exhibited signals of sequence conservation, with > 80% of sequence identity in > 50% of the alignment, regardless of whether they were found in homologous (76) or non-homologous (10) pseudochromosomes. In 51 instances, venom genes from an OG were present in *K. canariensis* but absent in *L. ventricosus*. Among these, 21 flanking regions located in homologous and seven in non-homologous pseudochromosomes showed > 80% of sequence identity in > 50% of the alignment, whereas the remaining cases displayed lower levels of sequence conservation (Supplementary Table S6). Conversely, in those cases where *L. ventricosus* had OGs not found in *K. canariensis*, 99 flanking regions located in homologous and five in non-homologous pseudochromosomes showed > 80% of sequence identity in > 50% of the alignment, whereas 87 cases were associated with more divergent flanking regions (Supplementary Table S6).

## Discussion

The BUSCO completeness scores for the three assembled venom gland transcriptomes of *K. canariensis* were within the range of 53–78%. While *de novo* assemblies merging RNA from several tissues and/or different developmental stages generally yield higher BUSCO scores [40], the transcriptomes here obtained showed completeness values similar or higher to those generally reported for transcriptomes derived from specialized tissues such as the venom gland [33] or the salivary gland [53] of other cones, but lower to those reported for venom glands of ants [54], spiders [55], pseudoscorpions [56] or snakes [57]. It is important to note that genes in the BUSCO databases are single-copy, mostly housekeeping genes that are not necessarily expressed in specialized tissues.

The number of transcripts encoded by venom genes was comparable to those reported for the venom gland transcriptomes of other species of cones (Supplementary Table S7). A high intraspecific variability in the expression of venom components was detected among the three individuals, as reported also in other cone species [20, 22, 23]. The overall low number of shared transcripts compared to the total number expressed in each venom gland is typical of cone snails, and has been associated to dietary shifts [17], physiological state [58], age/size [59], or geographical variability [60, 61], as well as stochastic expression differences.

The presence of representatives of the O1 and M superfamilies among the shared repertoire between the two species is consistent with findings in other cone snails, where these two superfamilies are among the most diverse and functionally relevant in their venom arsenals [13, 21, 23]. Members of the O1 superfamily encompass µ-, κ-, and ω-conotoxins that antagonize Na^+^, K^+^, and Ca²^+^ channels, respectively, as well as δ-conotoxins that block Na^+^ channel inactivation [19]. The M superfamily includes µ-conotoxins that target voltage-gated Na^+^ channels, κM-conotoxins that block voltage-gated K^+^ channels, and ψ-conotoxins acting as non-competitive antagonists of nicotinic acetylcholine receptors [19]. The diversity and conservation of the O1 and M superfamilies across individuals and species may indicate that they are essential functional components for prey capture and/or defense against predators. The identification of the B1 and Ggeo01 superfamilies among the shared transcripts between *K. canariensis* and *L. ventricosus* further highlights the existence of a minimum conserved core of essential venom components common to all cone species [21].

The number of venom genes in the *K. canariensis* genome is within the range reported for the genomes of *D. betulinus* (133; [25]), *C. textile* (199; [26]) and *L. ventricosus* (233; [13]). BLASTP analyses showed that the search with an e-value cutoff of 1.00E-04 rendered optimal results, as it was the one assigning the highest number of transcripts to genes, whereas lower thresholds started recovering other hits than venom genes, when manually checked. Given that the generated *K. canariensis* transcriptomic and genomic data successfully passed the diverse quality checks (e.g., sequencing depth, assembly completeness), the important discrepancy between number of venom genes (169) and that of transcripts (586) may have a biological basis. About 80% of the transcripts could be traced back to the genes and several molecular mechanisms could explain how the great diversity of transcripts could be generated. For instance, a smaller set of conotoxin genes may give rise to a much broader array of isoforms through alternative splicing. This is the case of GENK070 encoding conkunitzin 12, which presents two isoforms (see Supplementary Table S3) in the transcriptome, with same exons 1 and 3 but differing in the length of exon 2. Transcript diversity could also be generated through an increase of allelic variation at the population level [62, 63], which is reflected in the high variability among individuals detected in *K. canariensis*.

Of the complete venom genes in the genome, 70% in *K. canariens*is, 95% in *D. betulinus*, 64% in *C. textile*, and 91% in *L. ventricosus* corresponded to transcripts of the venom gland transcriptomes. While a rough 1:1 proportion of venom genes to transcripts was found in *D. betulinus* and *L. ventricosus*, no transcripts were detected for about 30% of the genes in *K. canariensis* and *C. textile*, as also reported for *Kioconus tribblei* [64], suggesting variable levels of transcriptional activity to generate final venom repertoires in the different species [65].

The genomes of *K. canariensis* [24] and *L. ventricosus* [13] showed large instances of macrosynteny, as well as evidence of an ancestral whole genome duplication in the caenogastropod lineage [13]. A similar pattern has been reported for the recently published genome of *C. textile* [26], although the sequence data for this genome is not yet available. Conotoxin precursor genes in *K. canariensis* and *L. ventricosus* were widely distributed throughout the genome and showed broadly similar patterns of chromosomal distribution and superfamily representation (Fig. 3; Supplementary Fig. S2), despite differences in total gene number. This pattern suggests either conserved mechanisms underlying venom gene diversification or relatively recent divergence in both species, as well as that the widespread distribution of venom genes in the genome is a conserved trait in cone snails. A scattered distribution of venom-related genes is also found in spider and snake genomes; however in snakes, some venom gene families have experienced several rounds of tandem gene duplication and are organized in arrays within a single chromosome [11, 14, 66]. Nevertheless, some striking differences in venom gene number and distribution between *K. canariensis* and *L. ventricosus* genomes were also detected, suggesting lineage-specific patterns of gene expansion or loss in the different gene families. To determine whether gene duplication in one species or loss in the other produced the patterns observed in each OG, new genomes need to be sequenced in order to perform phylogenetic and ancestral character-state analyses.

Overall, these patterns indicate that, beyond a shared core venom repertoire, each species has evolved a distinct set of auxiliary venom components. In contrast to *L. ventricosus* [13] and *C. textile* [26], the venom toolkit of *K. canariensis* is mostly dominated by representatives of a common core set with few extra OGs (i.e., members of OGs representing minor superfamilies are not found and larger superfamilies become less diverse; note that the possibility of missing highly divergent member cannot be completely discarded). Whether the reduced venom repertoire might indicate the smaller genome size of *K. canariensis*, a more specialized diet, or the insular condition of this endemic species remains to be tested.

When counterparts are found in both genomes, most OGs are retained directly from the ancestor within homologous chromosomes, suggesting that they are located within regions that have remained conserved since the divergence of the two species. Yet, in many cases, there are events of species-specific gene expansion (through local tandem duplication) or reduction. Similar mechanisms of localized gene turnover have been reported in other venomous taxa, including snakes and spiders, where tandem duplication (for expansion) and pseudogenization (for loss) cycles drive the rapid evolution of venom gene families [66–68].

Comparable patterns of expansion have been described not only for venom genes but also for multiple gene families across diverse taxa. For example, Membrane Intrinsic Proteins (MIPs) have undergone extensive duplication events across eukaryotes [69]. Likewise, the evolutionary history of molluscan lipopolysaccharide binding proteins and bactericidal permeability increasing proteins is notably complex, with independent and repeated duplication events occurring in parallel across several species [70]. In cone snails, chemosensory-related gene families (including G-protein-coupled receptors, ionotropic receptors, and gustatory receptors) also exhibit substantial duplication and high turnover rates, giving rise to clades conserved across protostomes, but also others species-specific [71]. Together, these examples highlight that local gene duplication is a widespread mechanism driving functional innovation. Overall, these observations are consistent with the Birth-and-Death model [72, 73], which provides a robust framework for understanding the dynamic evolution of large multigene families, and conotoxins in particular.

Finally, there are up to 16 instances of venom gene translocation (together with its flaking regions) to non-homologous pseudochromosomes, which may indicate that ectopic recombination, transposition, and large-scale chromosomal rearrangements (up to 33.14% of the venom genes were located in chromosomal inverted regions) could also contribute to the structural dynamism of venom repertoires (although the possibility that some apparent structural changes may reflect scaffolding/assembly artifacts cannot be fully discarded). These patterns are consistent with the hypothesis that venom gene families expand and reorganize dynamically [65, 74, 75]. Recombination processes underlying these rearrangements may be mediated by transposable elements (TEs), which have been shown to facilitate genomic reorganization, exon shuffling, and the diversification of gene families [26, 76, 77]. In *L. ventricosus*, most flanking regions of venom genes harbor Class I retrotransposons (Penelope, Gypsy, RTE elements) and Class II DNA transposons (Tc1-Mariner) [13], and could be influencing venom gene dynamics. However, whether there is a higher density of TEs in the flanking regions of venom genes than in the genomic background remains to be properly tested.

## Conclusions

Comparative genomic analyses among venomous species are crucial to understand the genetic basis of the evolutionary mechanisms that shape the origin and diversification of venoms. In this study, we integrated venom gland transcriptomics and comparative genomics between two closely related species of cone snails to shed light on the genetic basis and dynamics of toxin diversification in this group. Venom genes in the *K. canariensis* and *L. ventricosus* genomes were distributed across multiple pseudochromosomes and often surrounded by repetitive elements. A core set of venom genes common to both species was identified and an overall pattern of synteny across homologous pseudochromosomes was found. Yet, numerous lineage-specific venom loci as well as gene expansion/ contraction events were detected, reflecting dynamic evolutionary processes and adaptive flexibility shaping complete venom gene repertoires in cone snails. Besides local duplication as major driver of gene content divergence, the presence of venom loci within conserved flanking regions but located in non-homologous pseudochromosomes suggests that additional mechanisms, including segmental duplication, ectopic recombination, and transposition (many of which could be mediated by TEs), also contribute to the evolution and diversification of venom genes. The asymmetry in venom gene content between both genomes could be explained through gene expansion or loss events in any of the two species. Distinguishing between these alternative scenarios requires additional related species and a phylogenetic framework that allows us to infer ancestral states and directionality of change. Regardless of their origin, these patterns highlight the rapid turnover characteristic of conotoxin gene families and underscore the dynamic nature of venom gene evolution in cone snails.

## Supplementary Information

Supplementary Material 1.

## Data Availability

*K. canariensis* sequencing data are available at NCBI under BioProject PRJNA843968. RNA-sequencing raw reads for the three *K. canariensis* transcriptomes were deposited in the Sequence Read Archive (SRA-NCBI) and can be accessed under the following accession numbers: TF39: SRR36155560, TF42: SRR25393580, TF43: SRR25393579. All additional data supporting the conclusions of this article are available in the main text and/or the supplementary material.

## Abbreviations

HMM: Hidden Markov Model
NCBI: National Center for Biotechnology Information
OG: Orthogroup
PDI: Protein Disulfide Isomerases
TEs: Transposable Elements
