# Supplementary material for: Comparative analysis of venom genes in the chromosome-level genomes of two closely related cone snails
Source: BMC Genomics. 2026 Mar 18;27:409. doi: 10.1186/s12864-026-12745-4 (PMC13112664; doi:10.1186/s12864-026-12745-4)
Supplement: Supplementary file 1 — Supplementary Material 1. [file 12864_2026_12745_MOESM1_ESM.zip › SupplMattNew/SupplementaryFigures New.pdf]

Supplementary Figure S1. Distribution venom-related genes per pseudochromosome.

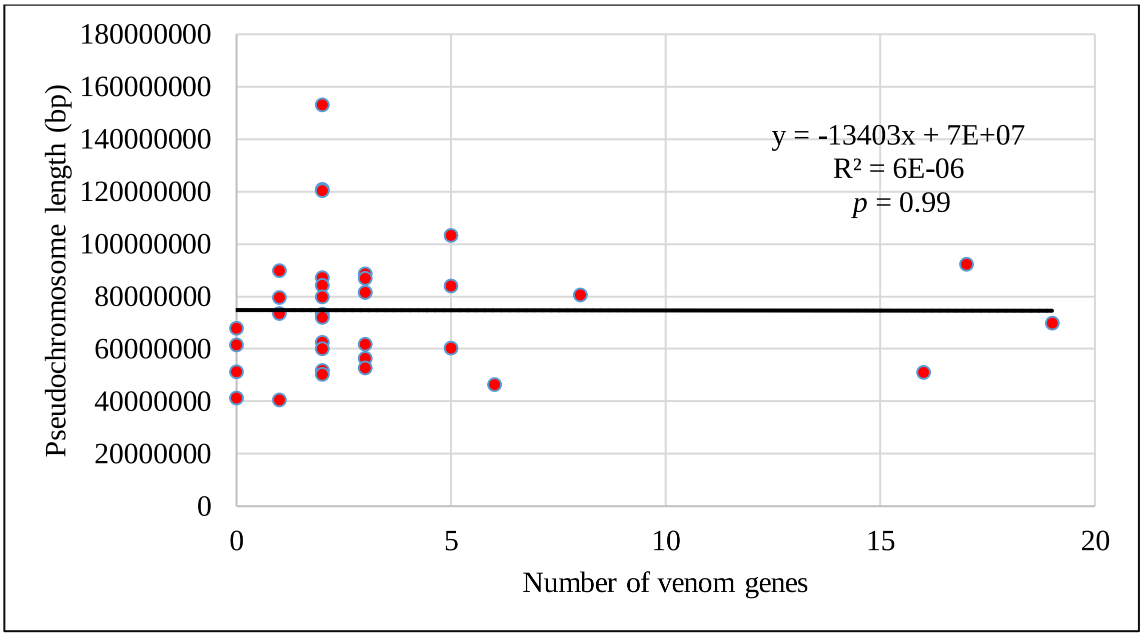

Supplementary Figure S2. Chromosomal localization of candidate venom genes in *K. canariensis* and *L. ventricosus*. Pairwise comparisons between homologous pseudochromosomes show differences in genomic distribution of these genes (in black) between *K. canariensis* and *L. ventricosus.* The vertical scale represents chromosome length.

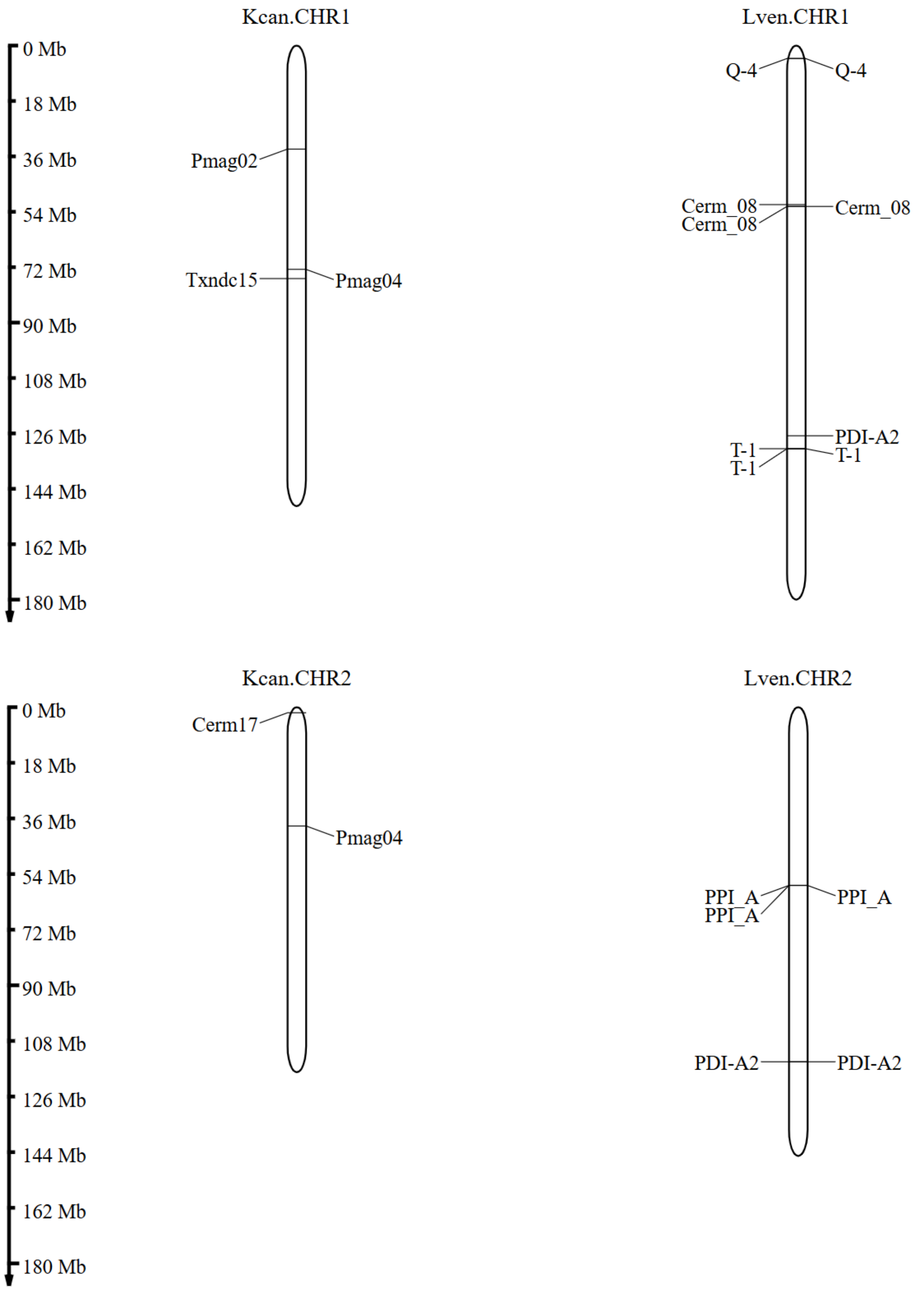

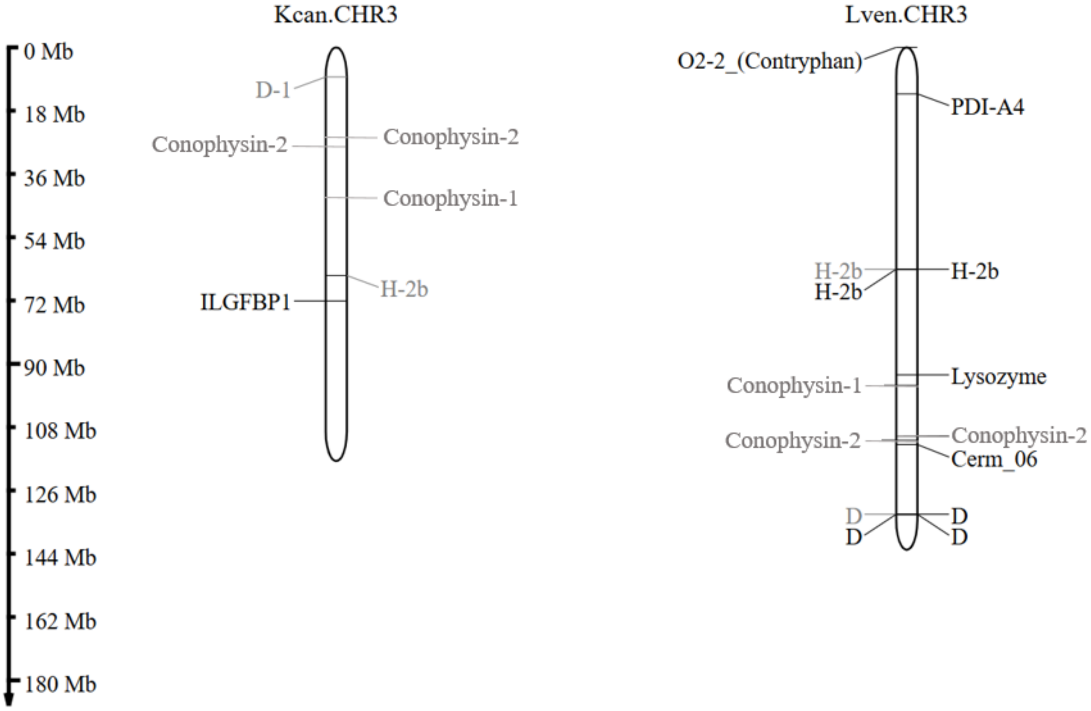
Kcan.CHR3
Lven.CHR3
0 Mb
18 Mb
36 Mb
54 Mb
72 Mb
90 Mb
108 Mb
126 Mb
144 Mb
162 Mb
180 Mb
D-1
Conophysin-2
Conophysin-2
Conophysin-1
H-2b
ILGFBP1
O2-2_(Contryphan)
PDI-A4
H-2b
H-2b
H-2b
Lysozyme
Conophysin-1
Conophysin-2
Conophysin-2
Cerm_06
D
D
D
D

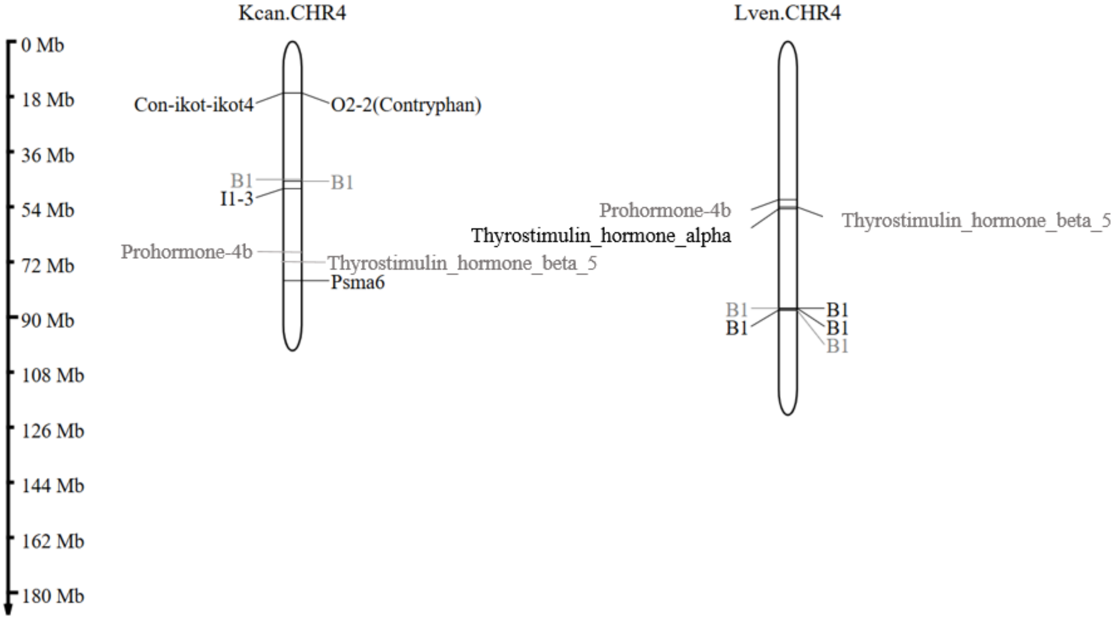
Kcan.CHR4
Lven.CHR4
0 Mb
18 Mb
36 Mb
54 Mb
72 Mb
90 Mb
108 Mb
126 Mb
144 Mb
162 Mb
180 Mb
Con-ikot-ikot4
O2-2(Contryphan)
B1
B1
I1-3
Prohormone-4b
Thyrostimulin_hormone_beta_5
Psma6
Prohormone-4b
Thyrostimulin_hormone_alpha
Thyrostimulin_hormone_beta_5
B1
B1
B1
B1
B1

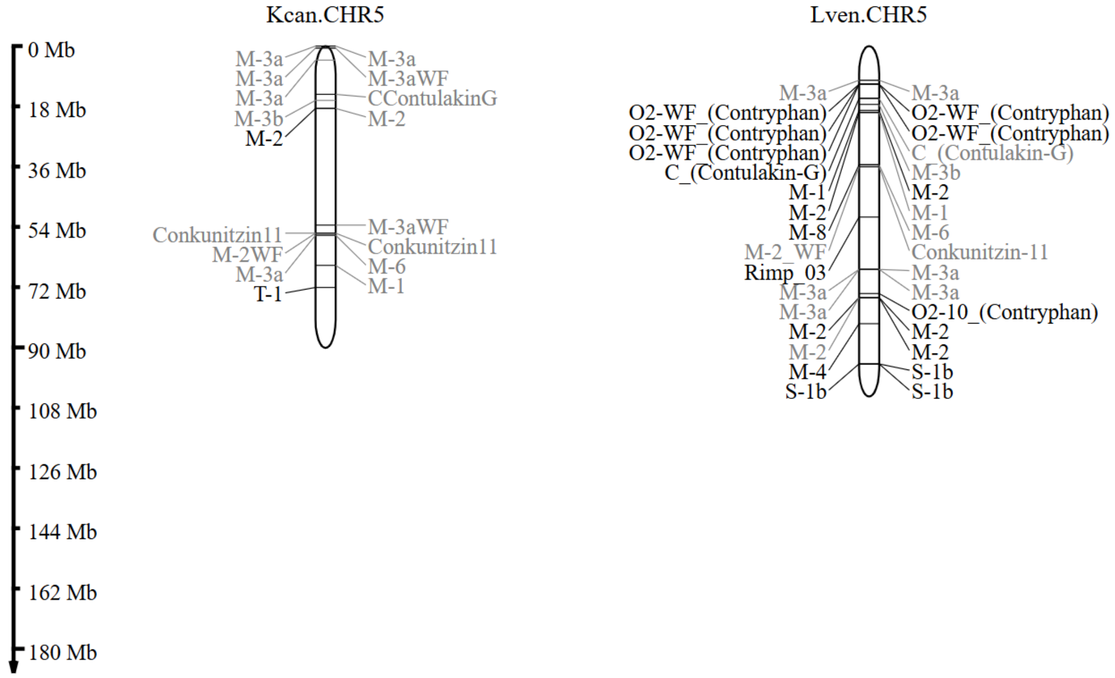
Kcan.CHR5
Lven.CHR5
0 Mb
18 Mb
36 Mb
54 Mb
72 Mb
90 Mb
108 Mb
126 Mb
144 Mb
162 Mb
180 Mb
M-3a
M-3a
M-3a
M-3b
M-2
M-3a
M-3aWF
CContulakinG
M-2
Conkunitzin11
M-2WF
M-3a
T-1
M-3aWF
Conkunitzin11
M-6
M-1
M-3a
O2-WF_(Contryphan)
O2-WF_(Contryphan)
O2-WF_(Contryphan)
C_(Contulakin-G)
M-1
M-2
M-8
M-2_WF
Rimp_03
M-3a
M-3a
M-2
M-2
M-4
S-1b
M-3a
O2-WF_(Contryphan)
O2-WF_(Contryphan)
C_(Contulakin-G)
M-3b
M-2
M-1
M-6
Conkunitzin-11
M-3a
M-3a
O2-10_(Contryphan)
M-2
M-2
S-1b
S-1b

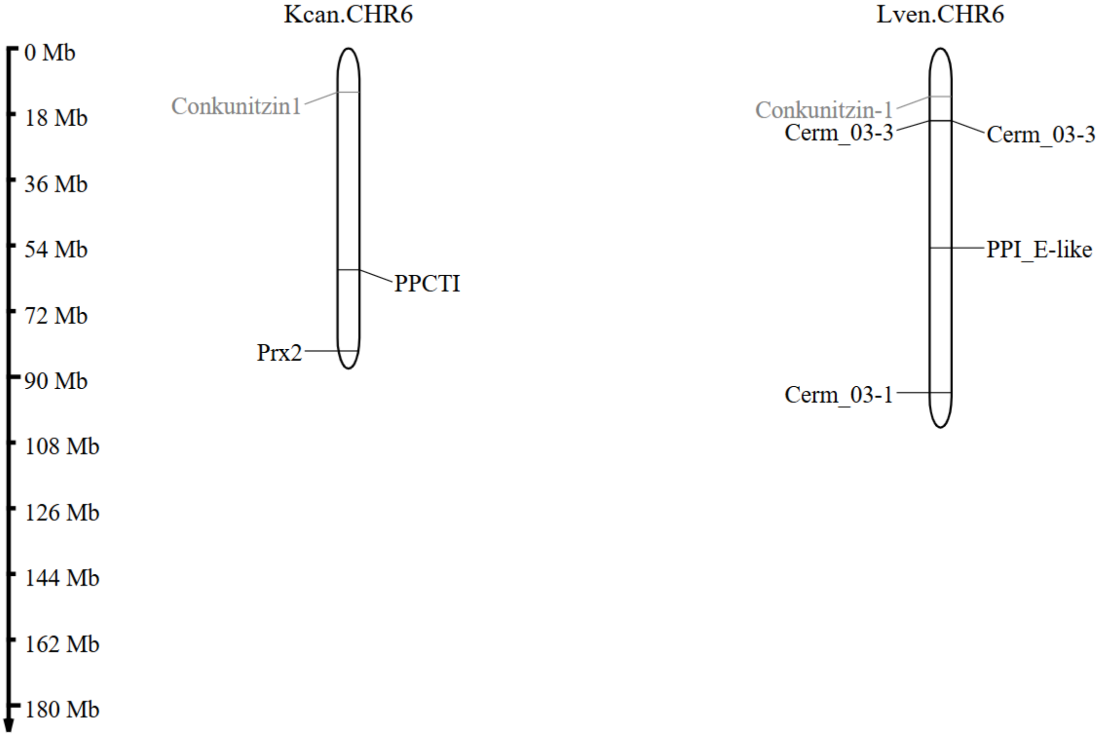
Kcan.CHR6
Lven.CHR6
0 Mb
18 Mb
36 Mb
54 Mb
72 Mb
90 Mb
108 Mb
126 Mb
144 Mb
162 Mb
180 Mb
Conkunitzin1
PPCTI
Prx2
Conkunitzin-1
Cerm_03-3
Cerm_03-3
PPI_E-like
Cerm_03-1

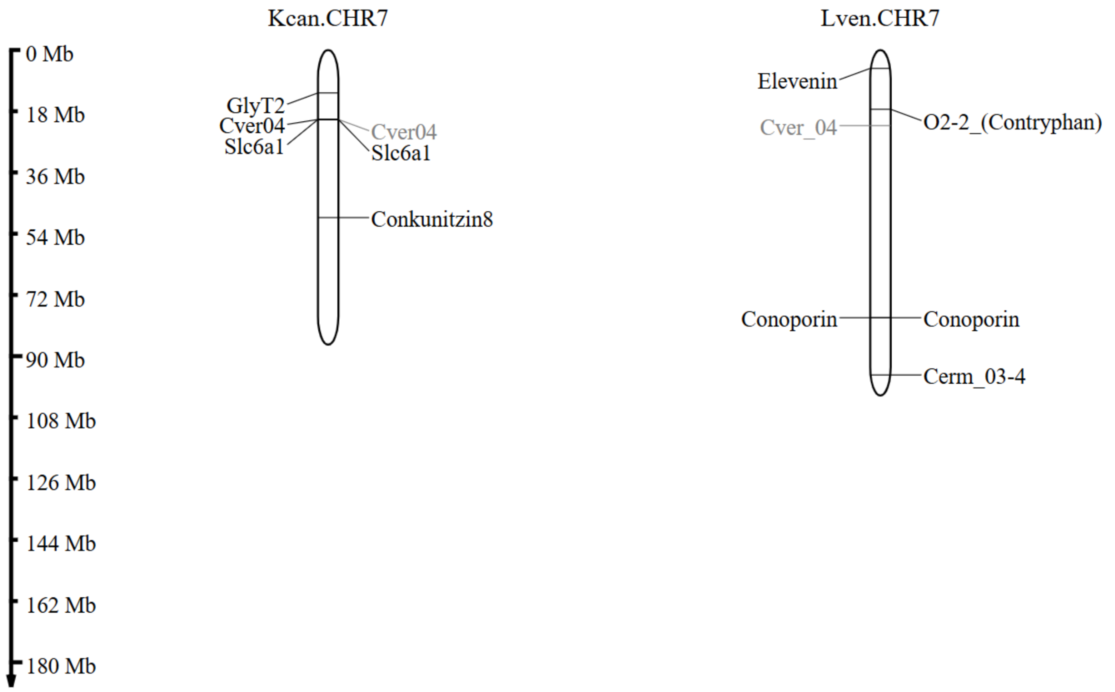
Kcan.CHR7
Lven.CHR7
0 Mb
18 Mb
36 Mb
54 Mb
72 Mb
90 Mb
108 Mb
126 Mb
144 Mb
162 Mb
180 Mb
GlyT2
Cver04
Slc6a1
Cver04
Slc6a1
Conkunitzin8
Elevenin
Cver_04
O2-2_(Contryphan)
Conoporin
Conoporin
Cerm_03-4

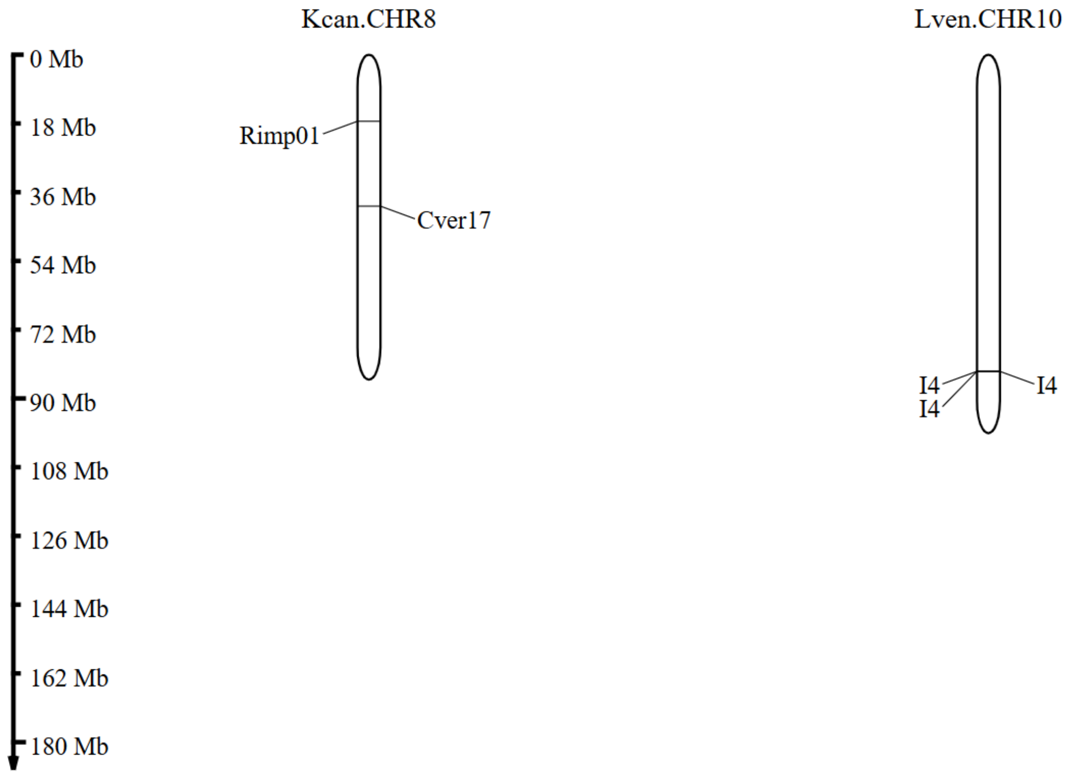
Kcan.CHR8
Lven.CHR10
0 Mb
18 Mb
36 Mb
54 Mb
72 Mb
90 Mb
108 Mb
126 Mb
144 Mb
162 Mb
180 Mb
Rimp01
Cver17
I4
I4
I4

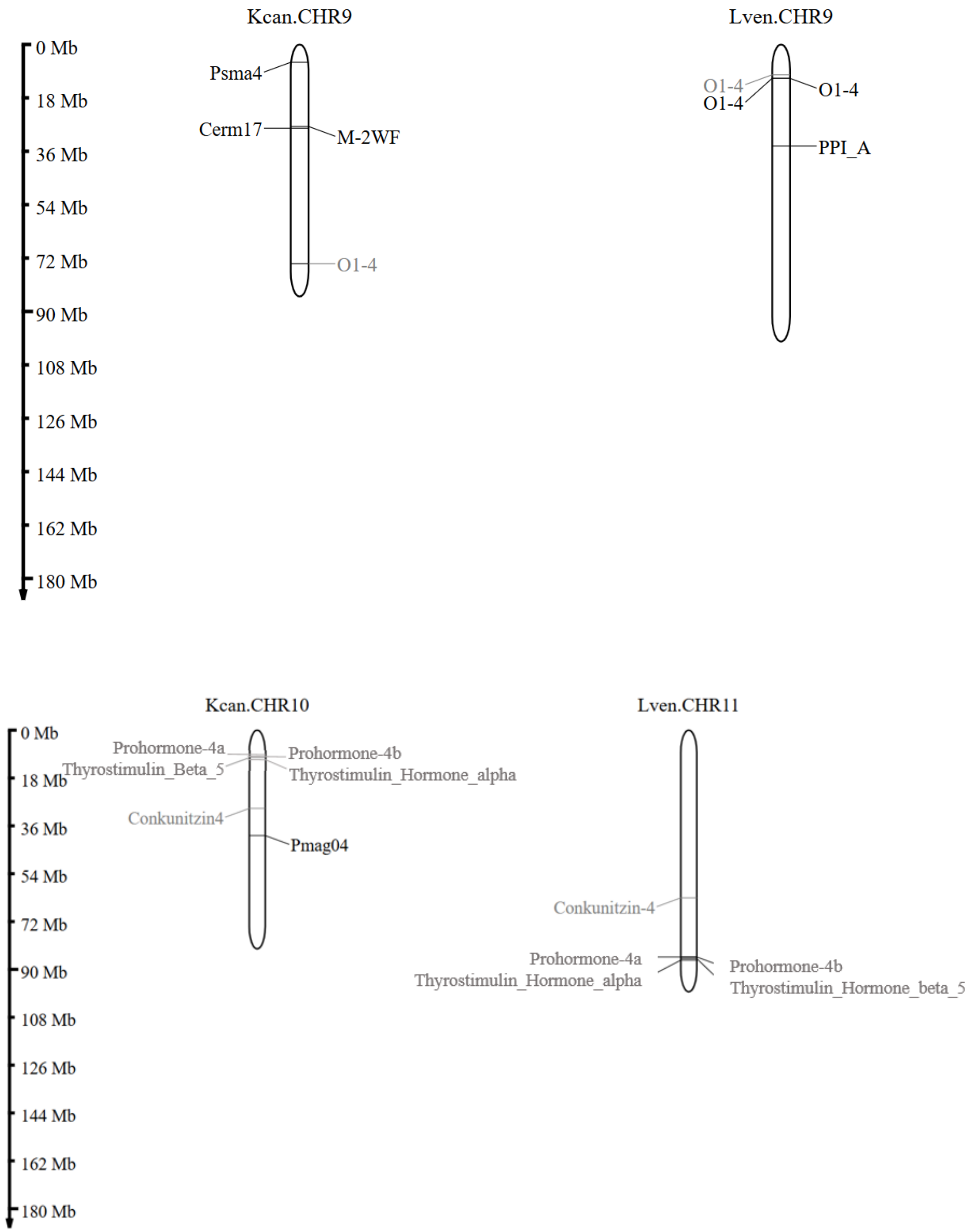
Kcan.CHR9
Lven.CHR9
0 Mb
18 Mb
36 Mb
54 Mb
72 Mb
90 Mb
108 Mb
126 Mb
144 Mb
162 Mb
180 Mb
Psma4
Cerm17
M-2WF
O1-4
O1-4
O1-4
O1-4
PPI_A
Kcan.CHR10
Lven.CHR11
Prohormone-4a
Thyrostimulin_Beta_5
Prohormone-4b
Thyrostimulin_Hormone_alpha
Conkunitzin4
Pmag04
Conkunitzin-4
Prohormone-4a
Thyrostimulin_Hormone_alpha
Prohormone-4b
Thyrostimulin_Hormone_beta_5

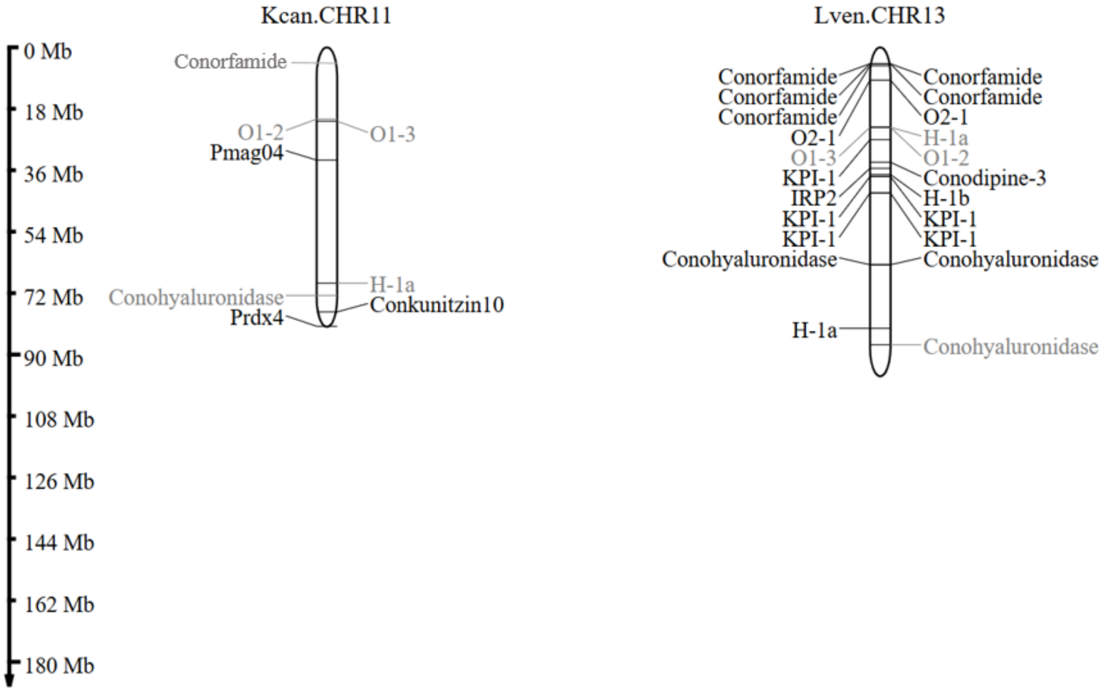
Kcan.CHR11
Lven.CHR13
0 Mb
18 Mb
36 Mb
54 Mb
72 Mb
90 Mb
108 Mb
126 Mb
144 Mb
162 Mb
180 Mb
Conorfamide
O1-2
O1-3
Pmag04
H-1a
Conohyaluronidase
Conkunitzin10
Prdx4
Conorfamide
Conorfamide
Conorfamide
O2-1
O1-3
KPI-1
IRP2
KPI-1
KPI-1
Conohyaluronidase
H-1a
Conorfamide
Conorfamide
O2-1
H-1a
O1-2
Conodipine-3
H-1b
KPI-1
KPI-1
Conohyaluronidase
Conohyaluronidase

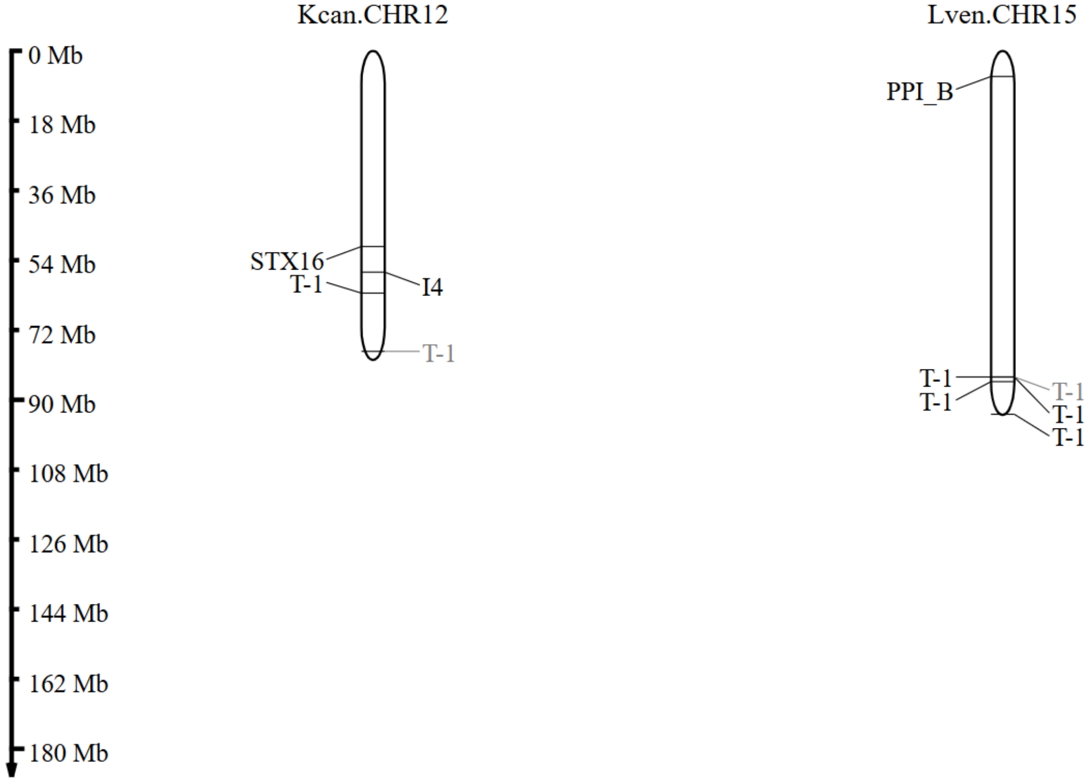
Kcan.CHR12
Lven.CHR15
0 Mb
18 Mb
36 Mb
54 Mb
72 Mb
90 Mb
108 Mb
126 Mb
144 Mb
162 Mb
180 Mb
STX16
T-1
I4
T-1
PPI_B
T-1
T-1
T-1
T-1
T-1

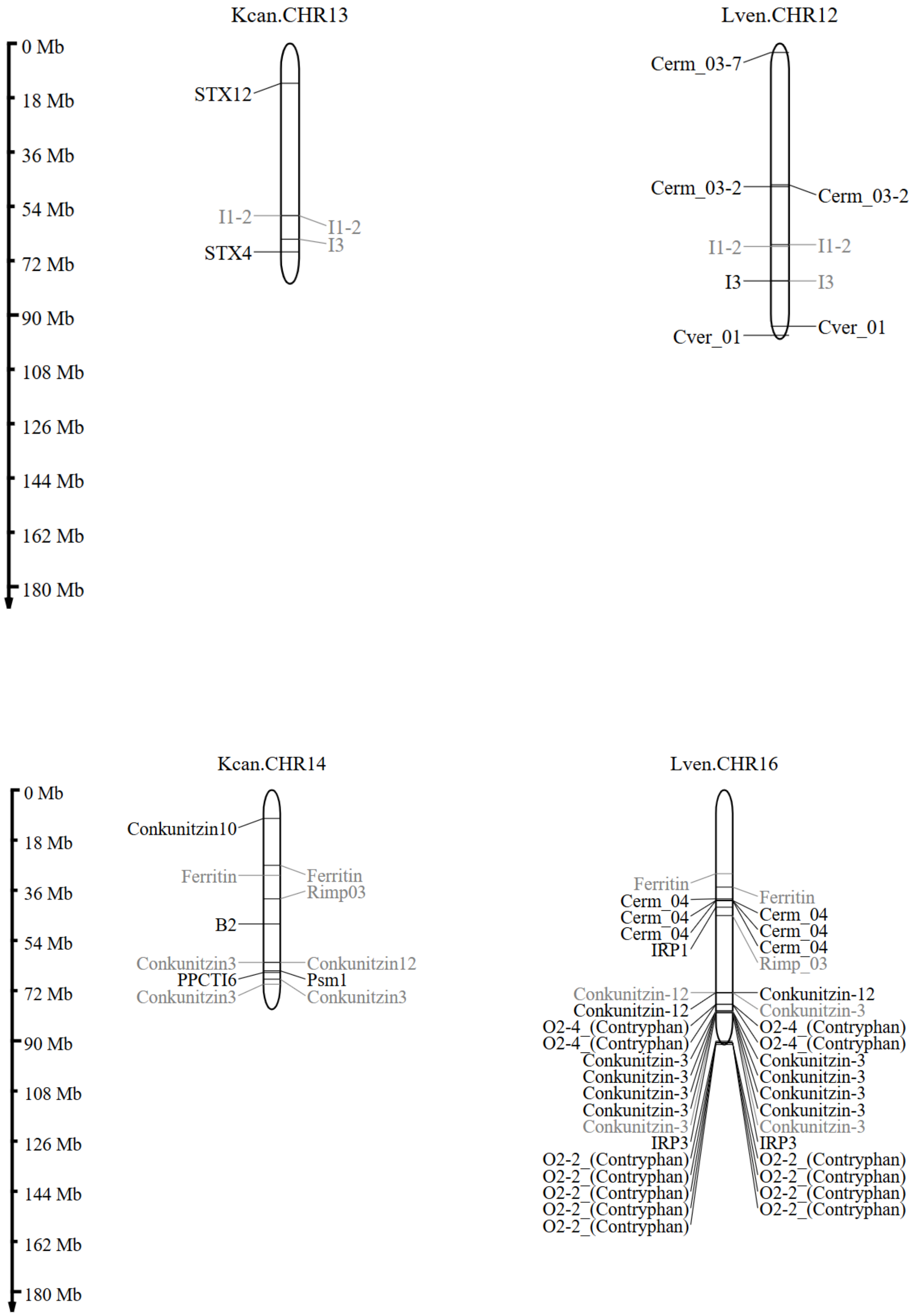
Kcan.CHR13
0 Mb
18 Mb
36 Mb
54 Mb
72 Mb
90 Mb
108 Mb
126 Mb
144 Mb
162 Mb
180 Mb
STX12
I1-2
I1-2
I3
STX4
Lven.CHR12
Cerm_03-7
Cerm_03-2
Cerm_03-2
I1-2
I1-2
I3
I3
Cver_01
Cver_01
Kcan.CHR14
0 Mb
18 Mb
36 Mb
54 Mb
72 Mb
90 Mb
108 Mb
126 Mb
144 Mb
162 Mb
180 Mb
Conkunitzin10
Ferritin
Ferritin
Rimp03
B2
Conkunitzin3
Conkunitzin12
PPCTI6
Psm1
Conkunitzin3
Conkunitzin3
Lven.CHR16
Ferritin
Cerm_04
Cerm_04
Cerm_04
IRP1
Conkunitzin-12
Conkunitzin-12
O2-4_(Contryphan)
O2-4_(Contryphan)
Conkunitzin-3
Conkunitzin-3
Conkunitzin-3
Conkunitzin-3
Conkunitzin-3
IRP3
O2-2_(Contryphan)
O2-2_(Contryphan)
O2-2_(Contryphan)
O2-2_(Contryphan)
O2-2_(Contryphan)
Ferritin
Cerm_04
Cerm_04
Cerm_04
Rimp_03
Conkunitzin-12
Conkunitzin-3
O2-4_(Contryphan)
O2-4_(Contryphan)
Conkunitzin-3
Conkunitzin-3
Conkunitzin-3
Conkunitzin-3
Conkunitzin-3
IRP3
O2-2_(Contryphan)
O2-2_(Contryphan)
O2-2_(Contryphan)
O2-2_(Contryphan)

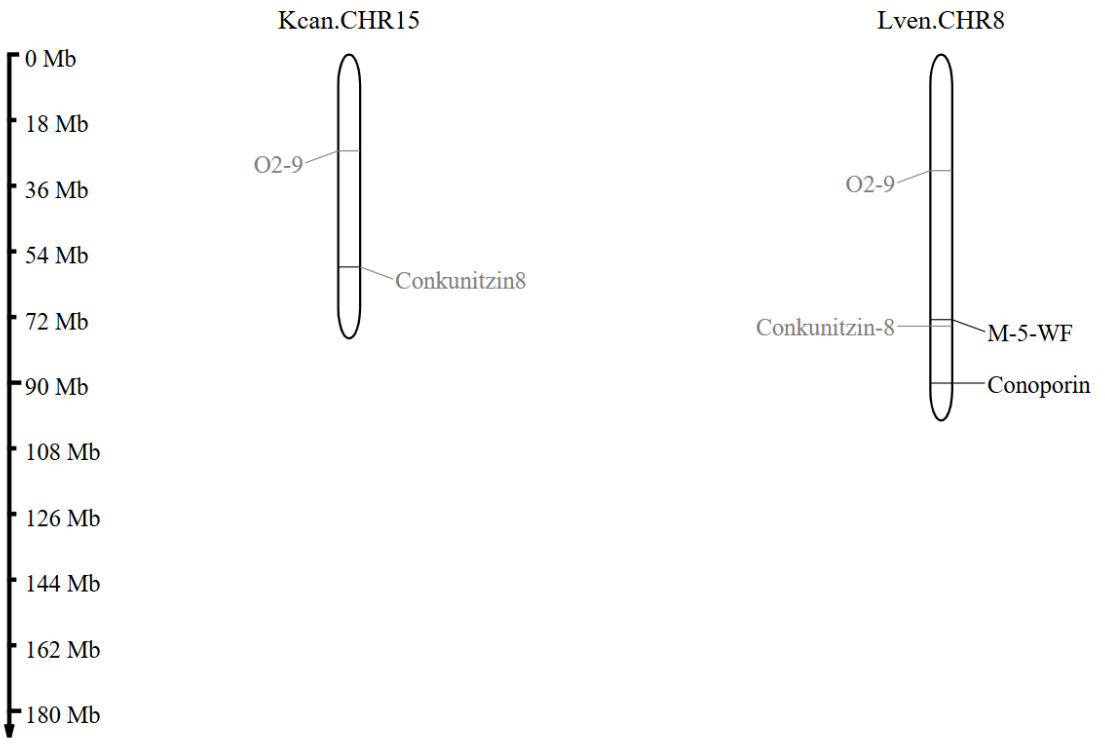
Kcan.CHR15
Lven.CHR8
0 Mb
18 Mb
36 Mb
54 Mb
72 Mb
90 Mb
108 Mb
126 Mb
144 Mb
162 Mb
180 Mb
O2-9
Conkunitzin8
O2-9
Conkunitzin-8
M-5-WF
Conoporin

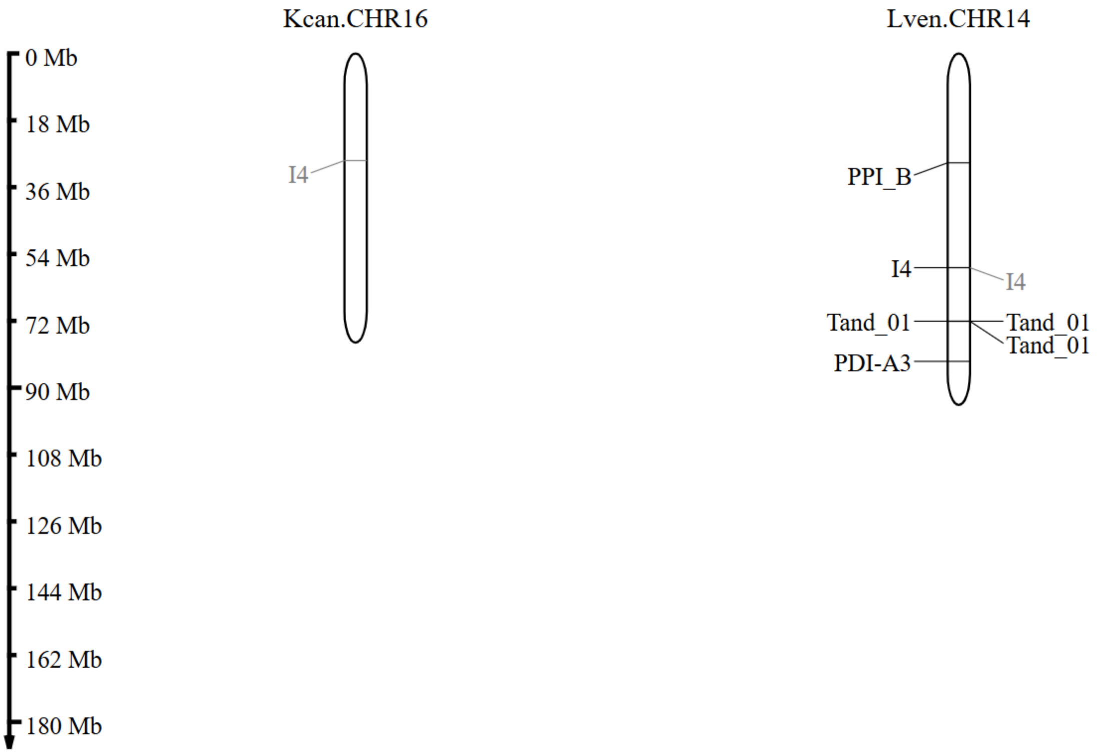
Kcan.CHR16
Lven.CHR14
0 Mb
18 Mb
36 Mb
54 Mb
72 Mb
90 Mb
108 Mb
126 Mb
144 Mb
162 Mb
180 Mb
I4
PPI_B
I4
I4
Tand_01
Tand_01
Tand_01
PDI-A3

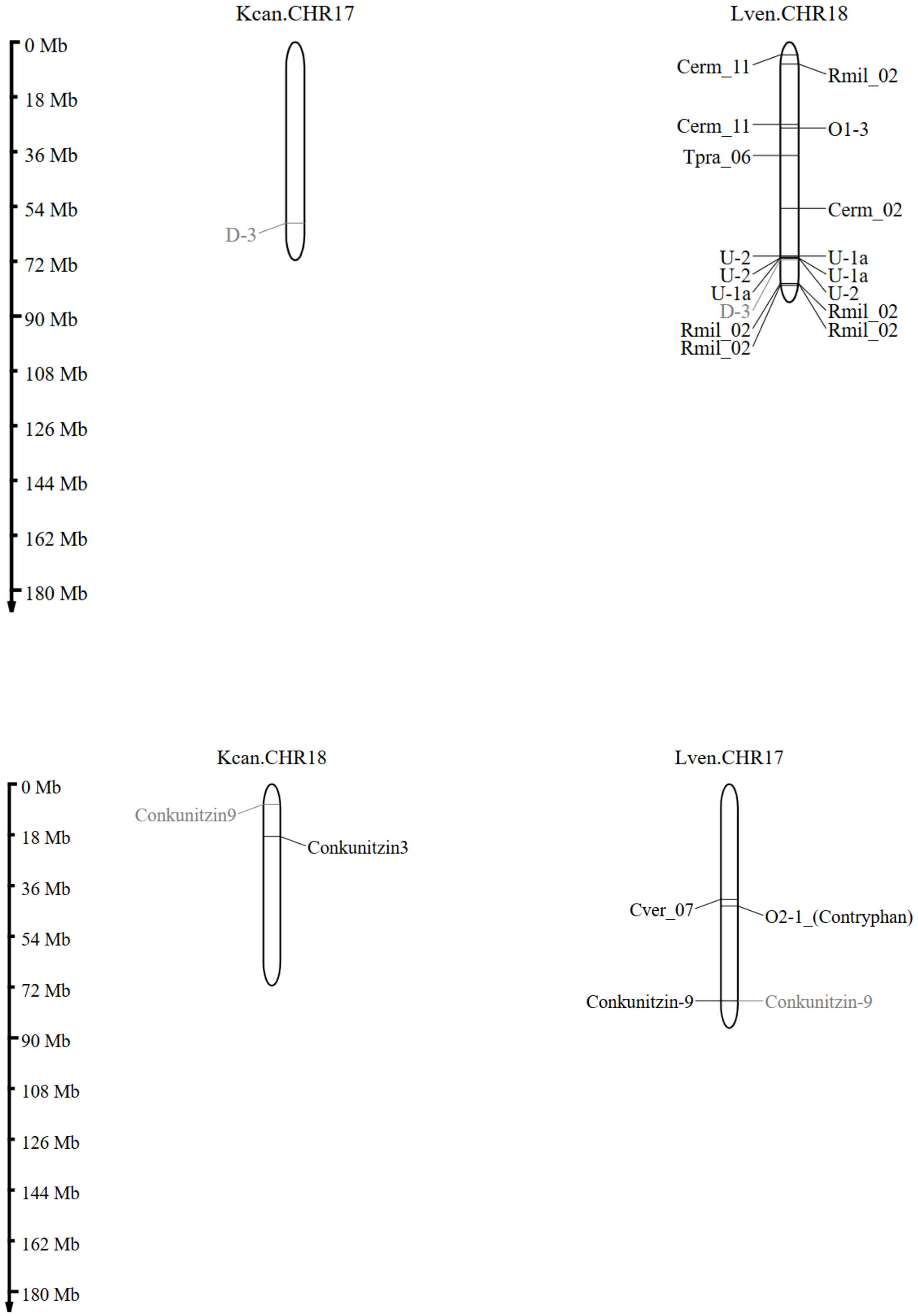

Kcan.CHR17
Lven.CHR18
0 Mb
18 Mb
36 Mb
54 Mb
72 Mb
90 Mb
108 Mb
126 Mb
144 Mb
162 Mb
180 Mb
D-3
Cerm_11
Rmil_02
Cerm_11
O1-3
Tpra_06
Cerm_02
U-2
U-2
U-1a
D-3
Rmil_02
Rmil_02
U-1a
U-1a
U-2
Rmil_02
Rmil_02
Kcan.CHR18
Lven.CHR17
0 Mb
18 Mb
36 Mb
54 Mb
72 Mb
90 Mb
108 Mb
126 Mb
144 Mb
162 Mb
180 Mb
Conkunitzin9
Conkunitzin3
Cver_07
O2-1_(Contryphan)
Conkunitzin-9
Conkunitzin-9

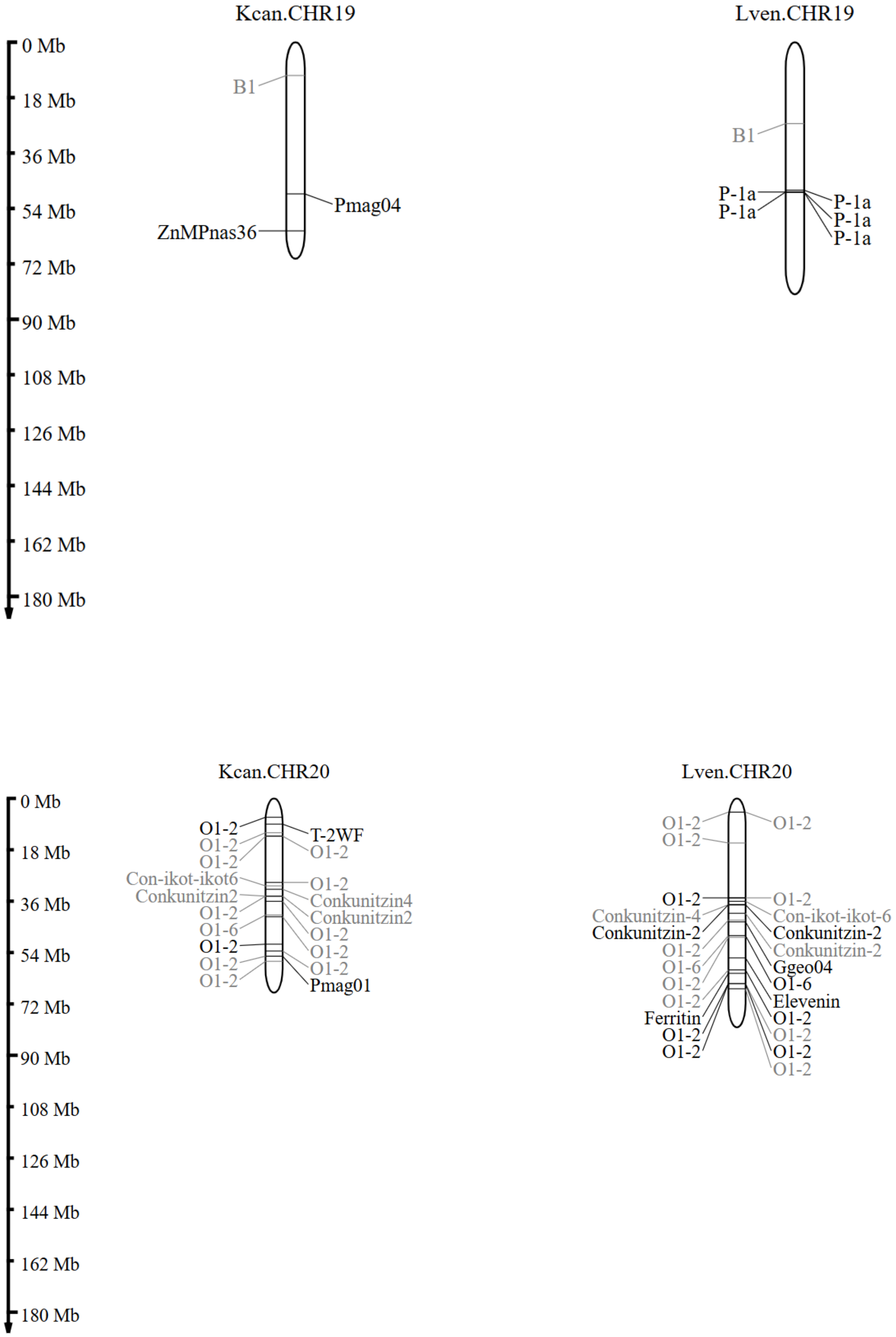

Kcan.CHR19
Lven.CHR19
0 Mb
18 Mb
36 Mb
54 Mb
72 Mb
90 Mb
108 Mb
126 Mb
144 Mb
162 Mb
180 Mb
B1
Pmag04
ZnMPnas36
B1
P-1a
P-1a
P-1a
P-1a
P-1a
Kcan.CHR20
Lven.CHR20
0 Mb
18 Mb
36 Mb
54 Mb
72 Mb
90 Mb
108 Mb
126 Mb
144 Mb
162 Mb
180 Mb
O1-2
O1-2
O1-2
Con-ikot-ikot6
Conkunitzin2
O1-2
O1-6
O1-2
O1-2
O1-2
T-2WF
O1-2
O1-2
Conkunitzin4
Conkunitzin2
O1-2
O1-2
O1-2
Pmag01
O1-2
O1-2
O1-2
Conkunitzin-4
Conkunitzin-2
O1-2
O1-6
O1-2
O1-2
Ferritin
O1-2
O1-2
O1-2
O1-2
Con-ikot-ikot-6
Conkunitzin-2
Conkunitzin-2
Ggeo04
O1-6
Elevenin
O1-2
O1-2
O1-2
O1-2

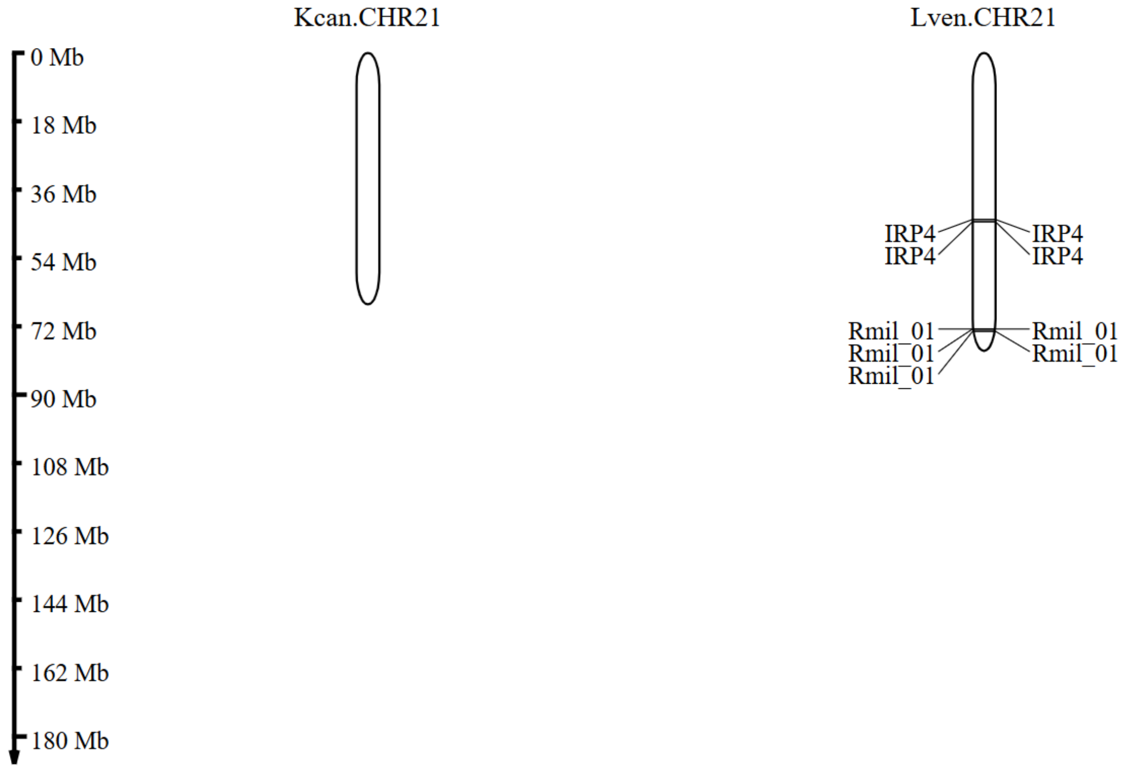
Kcan.CHR21
Lven.CHR21
0 Mb
18 Mb
36 Mb
54 Mb
72 Mb
90 Mb
108 Mb
126 Mb
144 Mb
162 Mb
180 Mb
IRP4
IRP4
IRP4
IRP4
Rmil_01
Rmil_01
Rmil_01
Rmil_01
Rmil_01

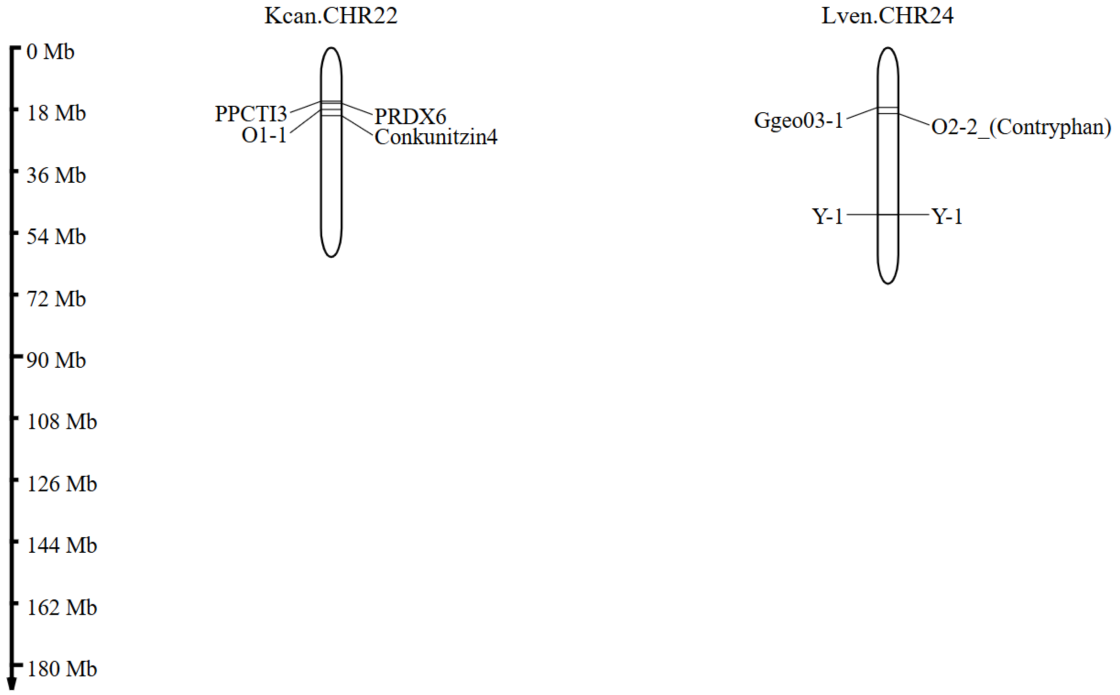
Kcan.CHR22
Lven.CHR24
0 Mb
18 Mb
36 Mb
54 Mb
72 Mb
90 Mb
108 Mb
126 Mb
144 Mb
162 Mb
180 Mb
PPCTI3
O1-1
PRDX6
Conkunitzin4
Ggeo03-1
O2-2_(Contryphan)
Y-1
Y-1

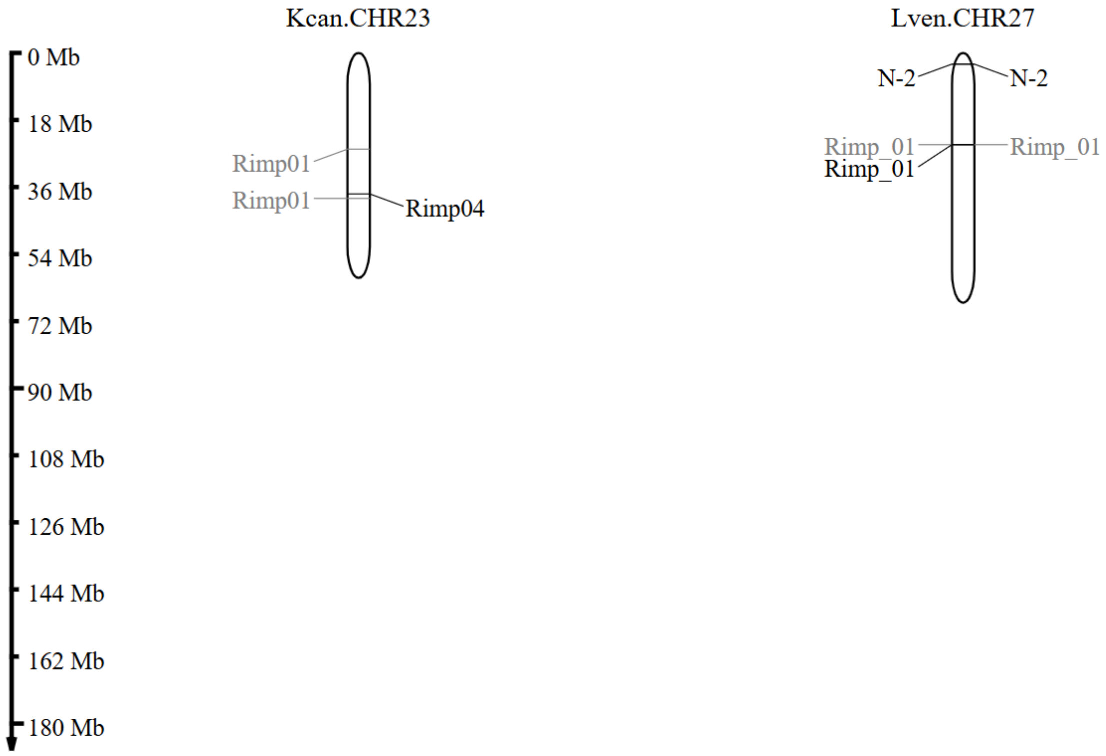
Kcan.CHR23
Lven.CHR27
0 Mb
18 Mb
36 Mb
54 Mb
72 Mb
90 Mb
108 Mb
126 Mb
144 Mb
162 Mb
180 Mb
Rimp01
Rimp01
Rimp04
N-2
N-2
Rimp_01
Rimp_01
Rimp_01

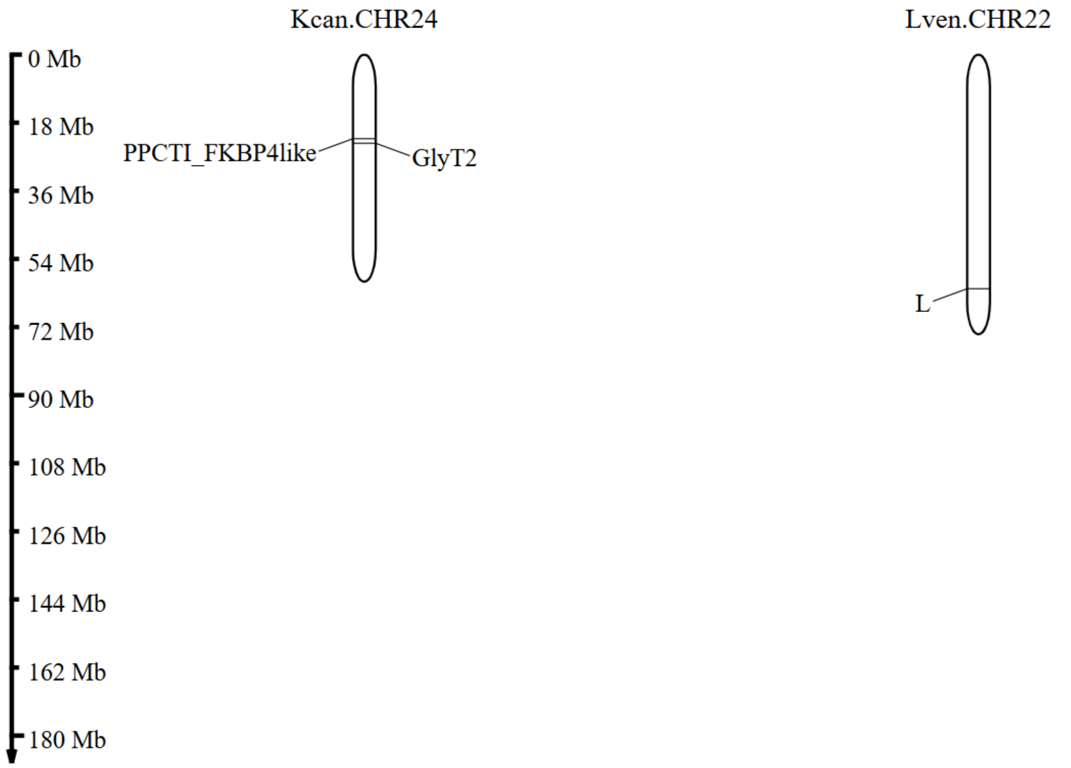
Kcan.CHR24
Lven.CHR22
0 Mb
18 Mb
36 Mb
54 Mb
72 Mb
90 Mb
108 Mb
126 Mb
144 Mb
162 Mb
180 Mb
PPCTI_FKBP4like
GlyT2
L

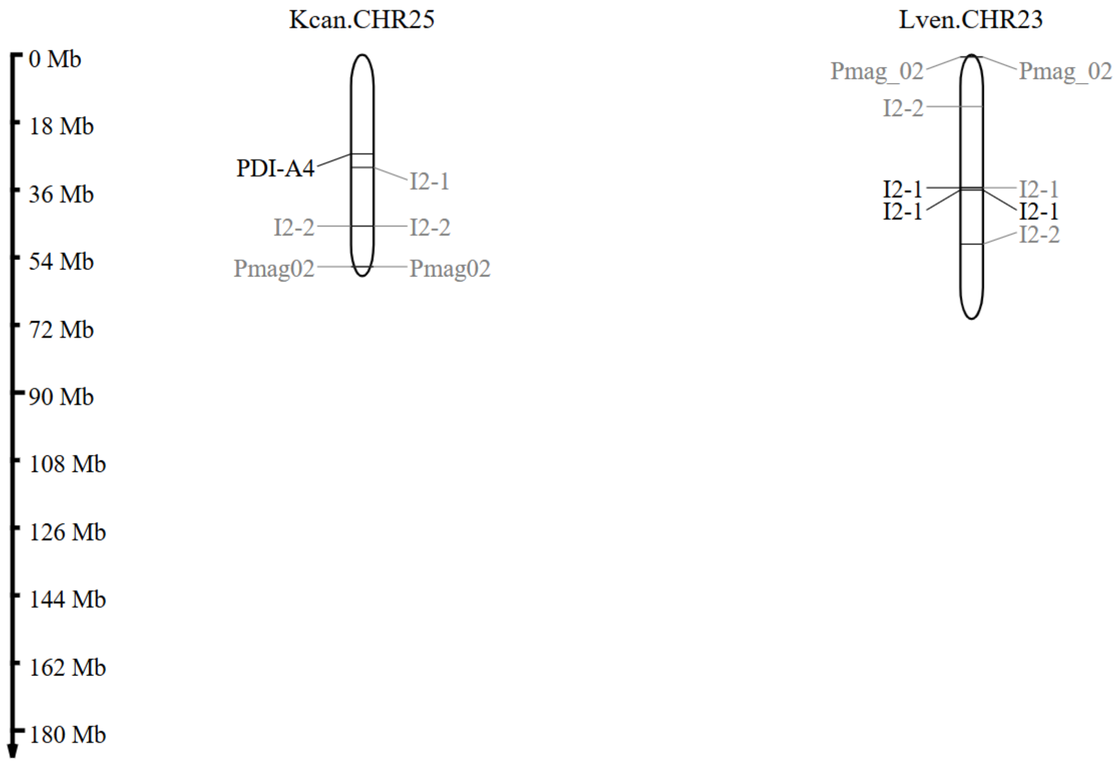
Kcan.CHR25
Lven.CHR23
0 Mb
18 Mb
36 Mb
54 Mb
72 Mb
90 Mb
108 Mb
126 Mb
144 Mb
162 Mb
180 Mb
PDI-A4
I2-1
I2-2
I2-2
Pmag02
Pmag02
Pmag_02
Pmag_02
I2-2
I2-1
I2-1
I2-1
I2-1
I2-2

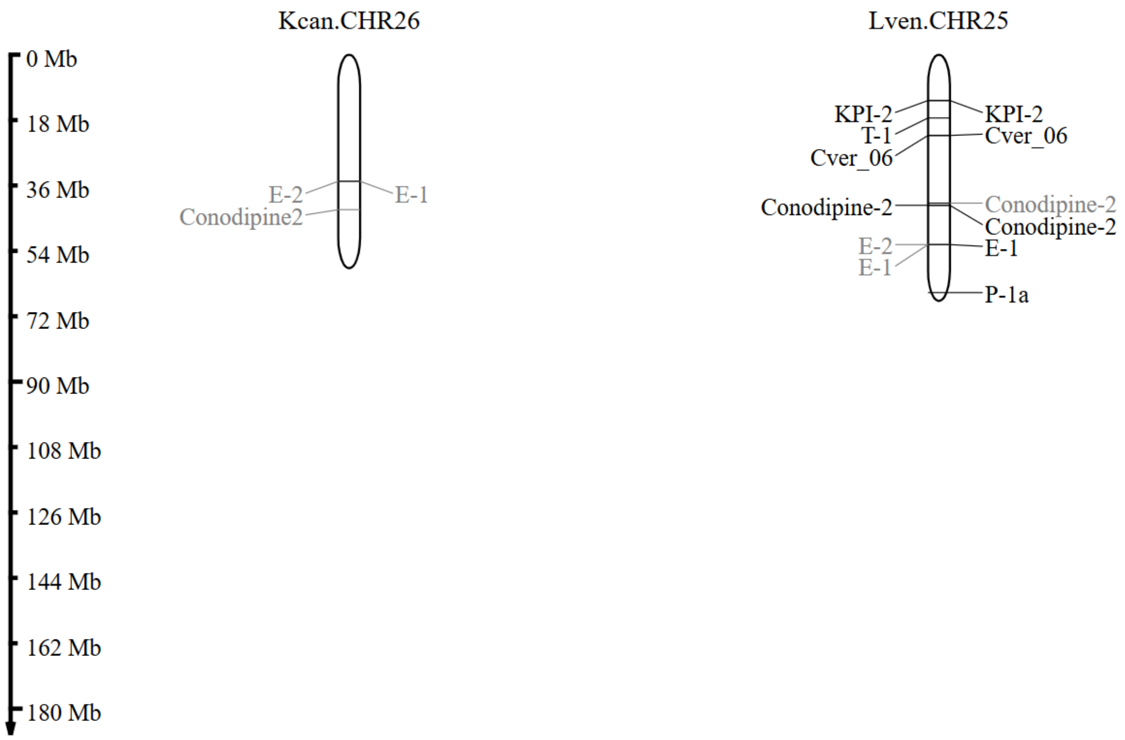
Kcan.CHR26
Lven.CHR25
0 Mb
18 Mb
36 Mb
54 Mb
72 Mb
90 Mb
108 Mb
126 Mb
144 Mb
162 Mb
180 Mb
E-2
Conodipine2
E-1
KPI-2
T-1
Cver_06
KPI-2
Cver_06
Conodipine-2
Conodipine-2
Conodipine-2
E-2
E-1
E-1
P-1a

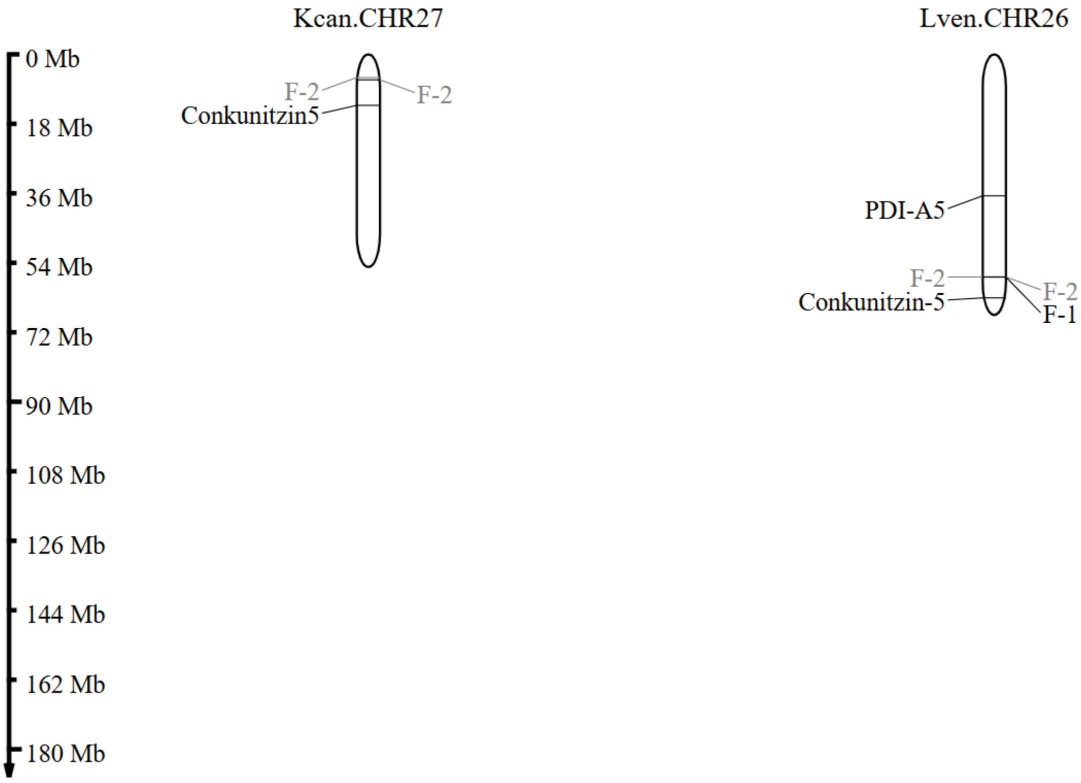
Kcan.CHR27
Lven.CHR26
0 Mb
18 Mb
36 Mb
54 Mb
72 Mb
90 Mb
108 Mb
126 Mb
144 Mb
162 Mb
180 Mb
F-2
Conkunitzin5
F-2
PDI-A5
F-2
Conkunitzin-5
F-2
F-1

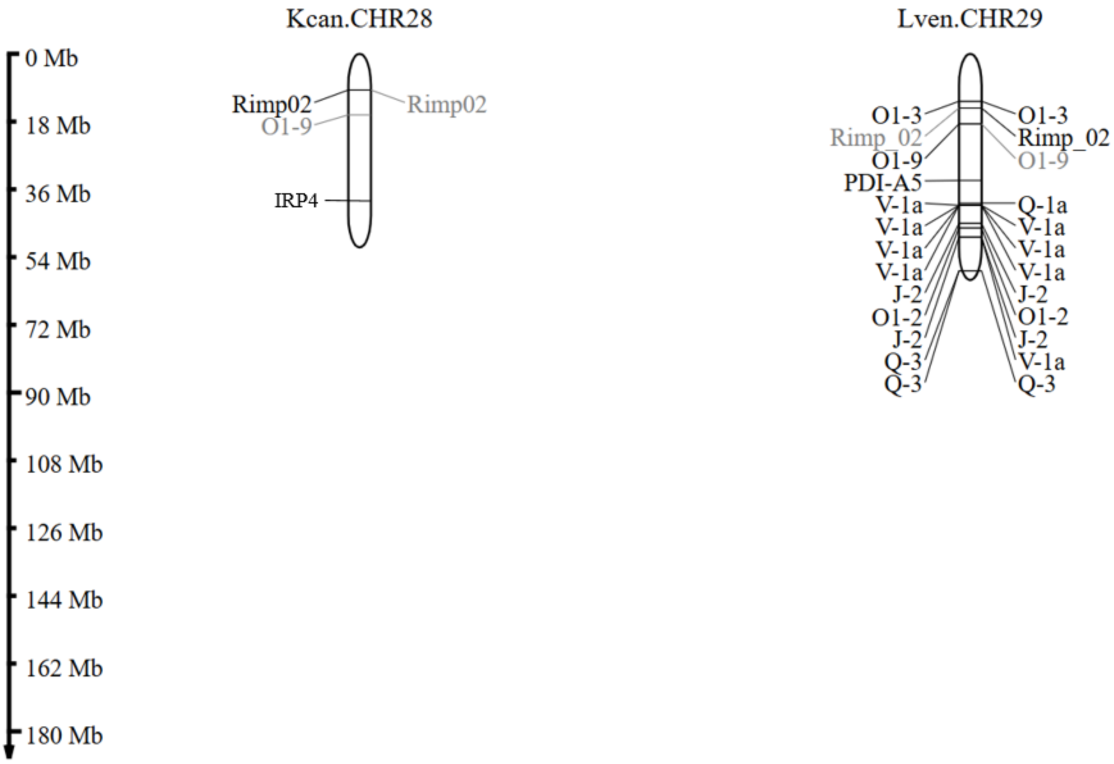
Kcan.CHR28
Lven.CHR29
0 Mb
18 Mb
36 Mb
54 Mb
72 Mb
90 Mb
108 Mb
126 Mb
144 Mb
162 Mb
180 Mb
Rimp02
O1-9
IRP4
Rimp02
O1-3
Rimp_02
O1-9
PDI-A5
V-1a
V-1a
V-1a
V-1a
J-2
O1-2
J-2
Q-3
Q-3
O1-3
Rimp_02
O1-9
Q-1a
V-1a
V-1a
V-1a
J-2
O1-2
J-2
V-1a
Q-3

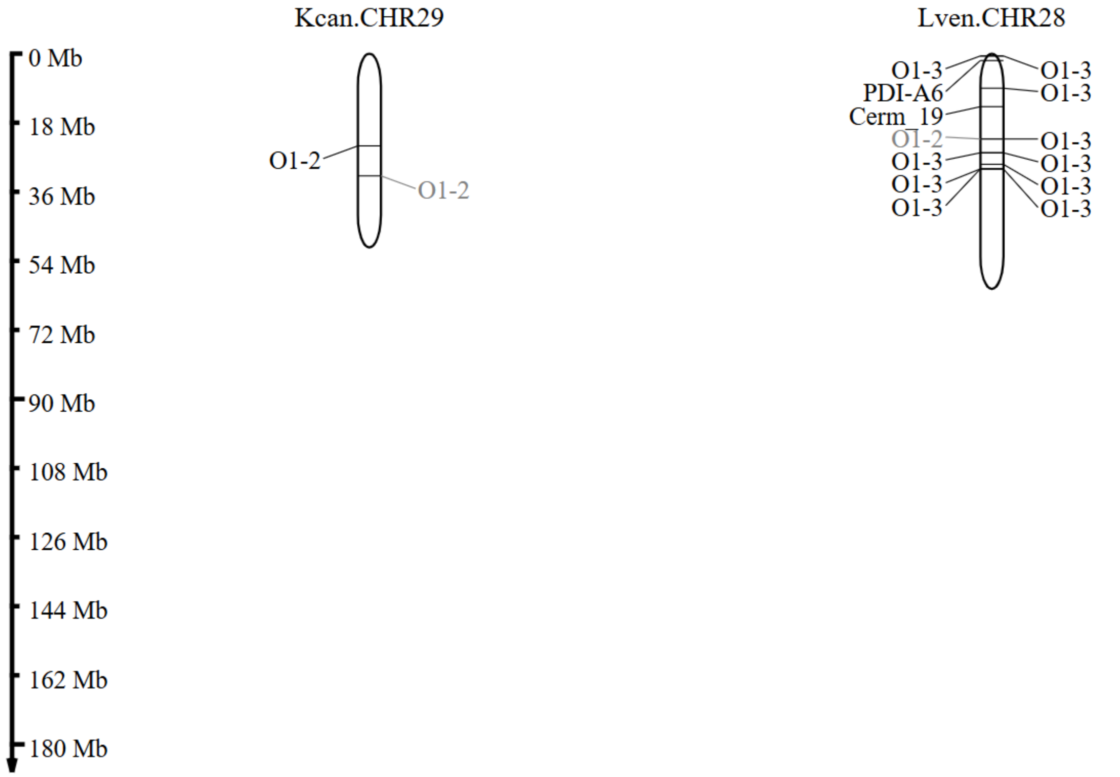
Kcan.CHR29
Lven.CHR28
0 Mb
18 Mb
36 Mb
54 Mb
72 Mb
90 Mb
108 Mb
126 Mb
144 Mb
162 Mb
180 Mb
O1-2
O1-2
O1-3
PDI-A6
Cerm_19
O1-2
O1-3
O1-3
O1-3
O1-3
O1-3
O1-3
O1-3
O1-3
O1-3

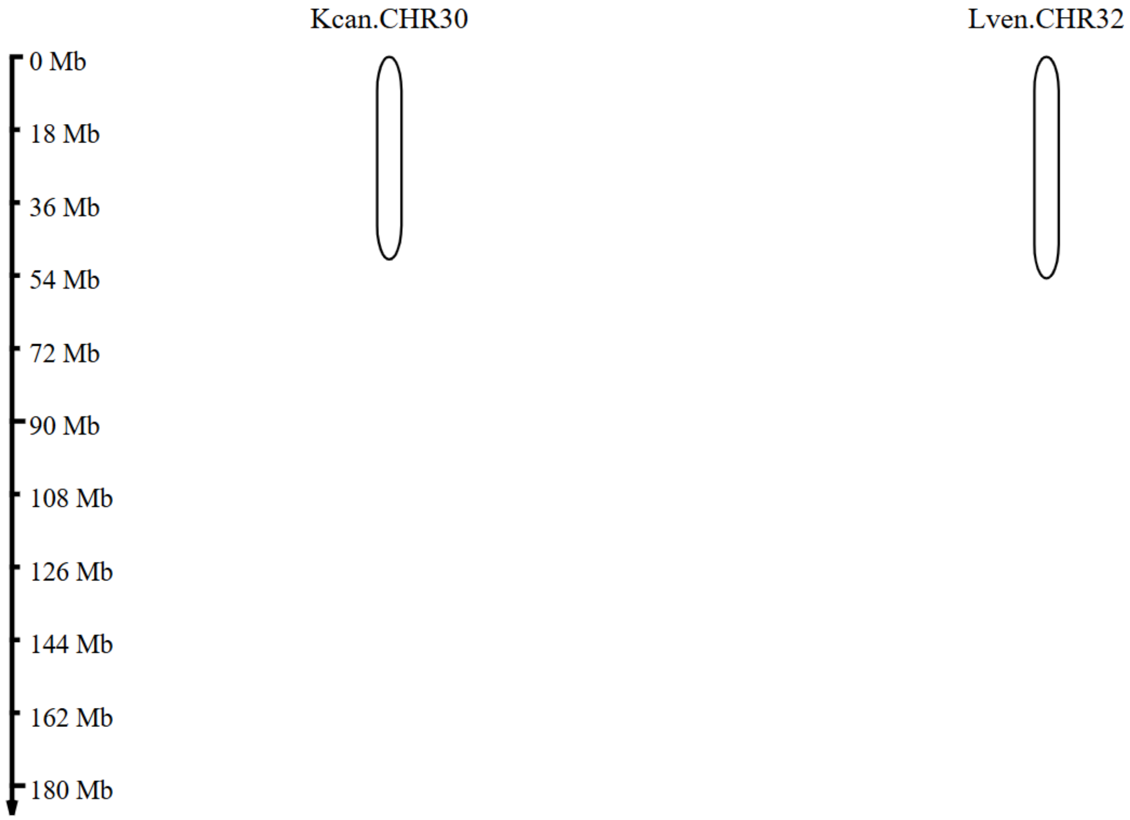
Kcan.CHR30
Lven.CHR32
0 Mb
18 Mb
36 Mb
54 Mb
72 Mb
90 Mb
108 Mb
126 Mb
144 Mb
162 Mb
180 Mb

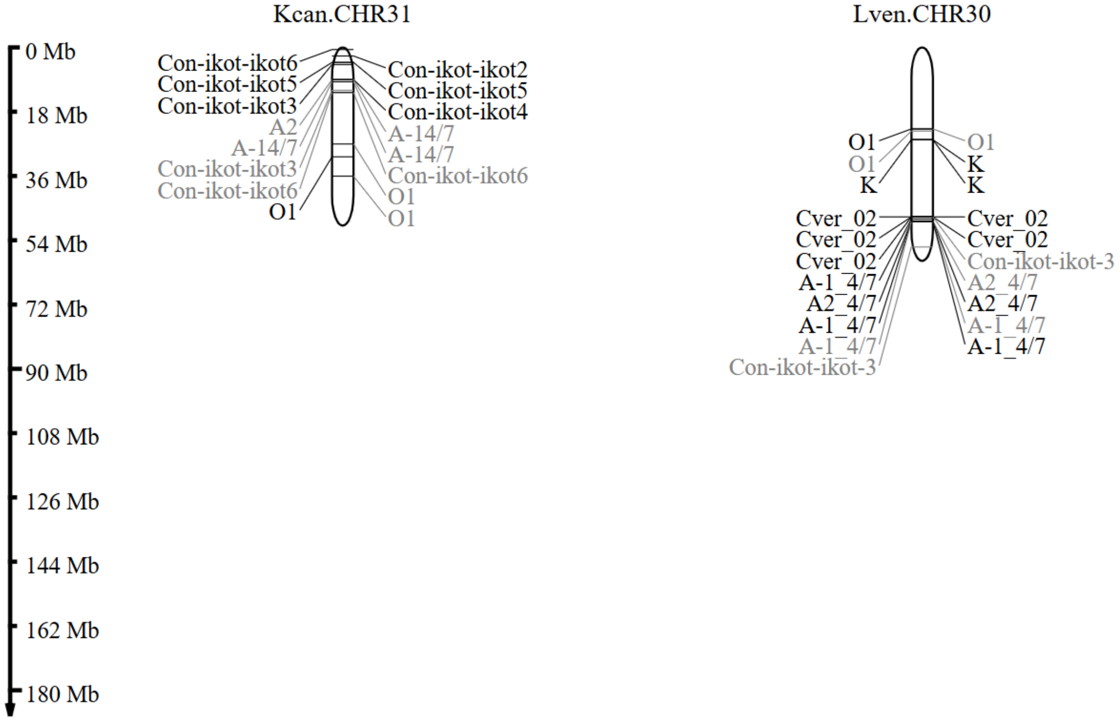
Kcan.CHR31
Lven.CHR30
0 Mb
18 Mb
36 Mb
54 Mb
72 Mb
90 Mb
108 Mb
126 Mb
144 Mb
162 Mb
180 Mb
Con-ikot-ikot6
Con-ikot-ikot5
Con-ikot-ikot3
A2
A-14/7
Con-ikot-ikot3
Con-ikot-ikot6
O1
Con-ikot-ikot2
Con-ikot-ikot5
Con-ikot-ikot4
A-14/7
A-14/7
Con-ikot-ikot6
O1
O1
O1
O1
K
O1
K
K
Cver_02
Cver_02
Cver_02
A-1_4/7
A2_4/7
A-1_4/7
A-1_4/7
Con-ikot-ikot-3
Cver_02
Cver_02
Con-ikot-ikot-3
A2_4/7
A2_4/7
A-1_4/7
A-1_4/7

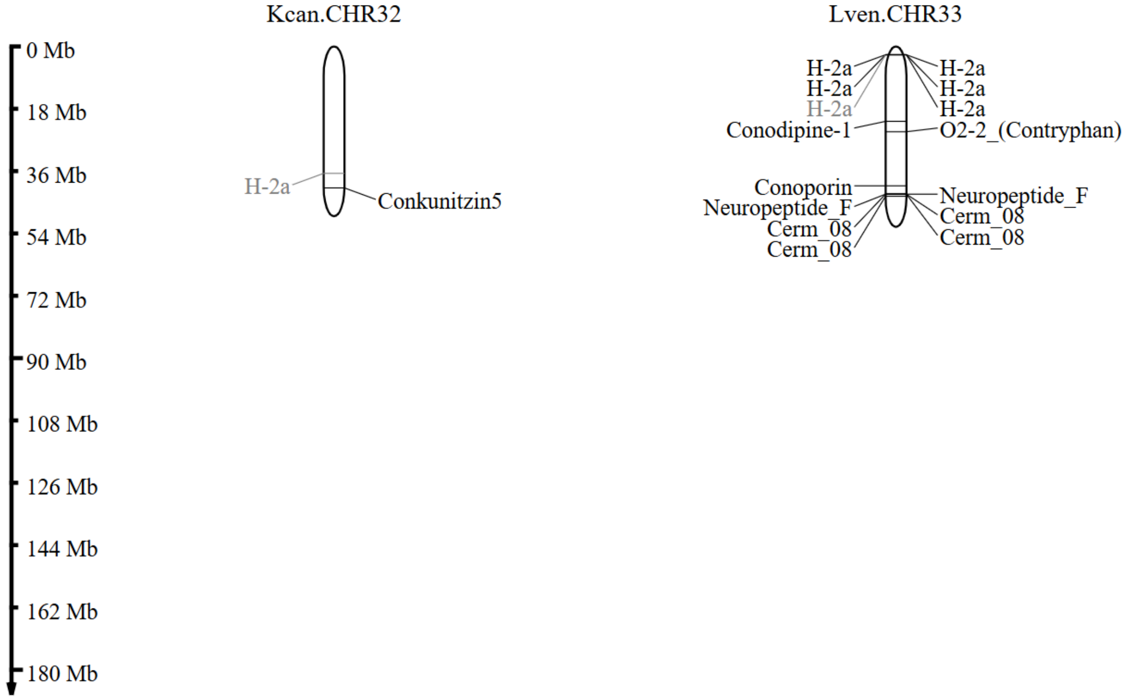
Kcan.CHR32
Lven.CHR33
0 Mb
18 Mb
36 Mb
54 Mb
72 Mb
90 Mb
108 Mb
126 Mb
144 Mb
162 Mb
180 Mb
H-2a
Conkunitzin5
H-2a
H-2a
H-2a
Conodipine-1
Conoporin
Neuropeptide_F
Cerm_08
Cerm_08
H-2a
H-2a
H-2a
O2-2_(Contryphan)
Neuropeptide_F
Cerm_08
Cerm_08

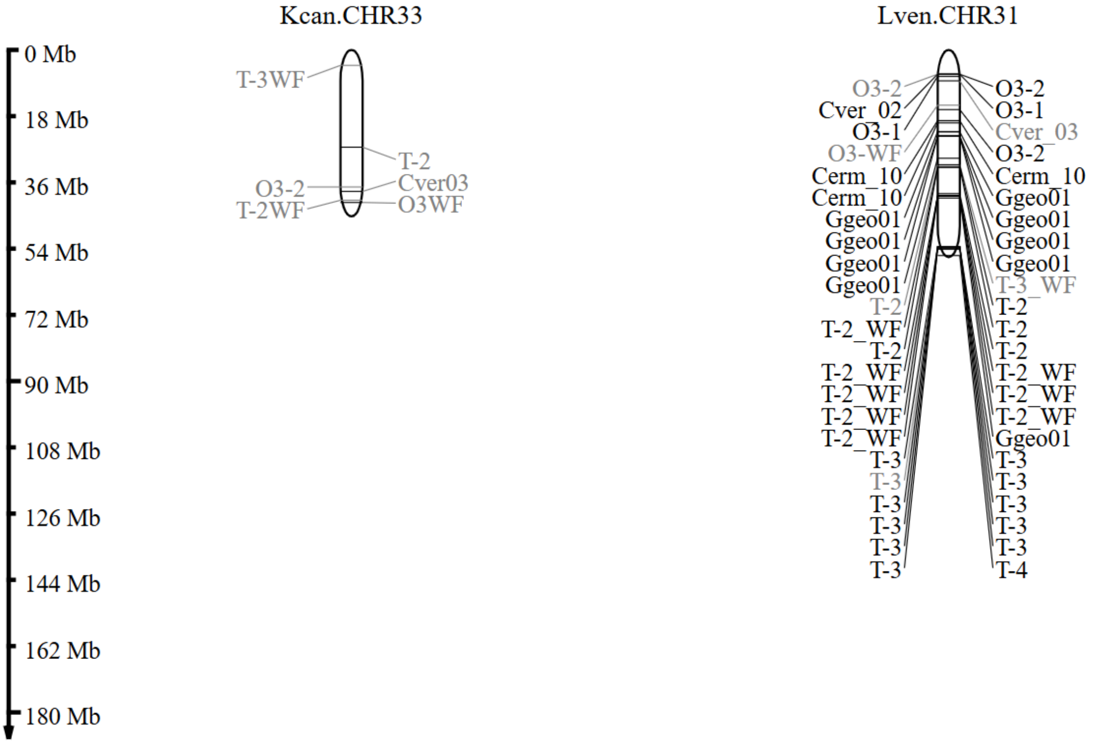
Kcan.CHR33
Lven.CHR31
0 Mb
18 Mb
36 Mb
54 Mb
72 Mb
90 Mb
108 Mb
126 Mb
144 Mb
162 Mb
180 Mb
T-3WF
T-2
O3-2
Cver03
T-2WF
O3WF
O3-2
Cver_02
O3-1
O3-WF
Cerm_10
Cerm_10
Ggeo01
Ggeo01
Ggeo01
Ggeo01
T-2
T-2_WF
T-2
T-2_WF
T-2_WF
T-2_WF
T-2_WF
T-3
T-3
T-3
T-3
T-3
T-3
O3-2
O3-1
Cver_03
O3-2
Cerm_10
Ggeo01
Ggeo01
Ggeo01
Ggeo01
T-3_WF
T-2
T-2
T-2
T-2_WF
T-2_WF
T-2_WF
Ggeo01
T-3
T-3
T-3
T-3
T-3
T-4

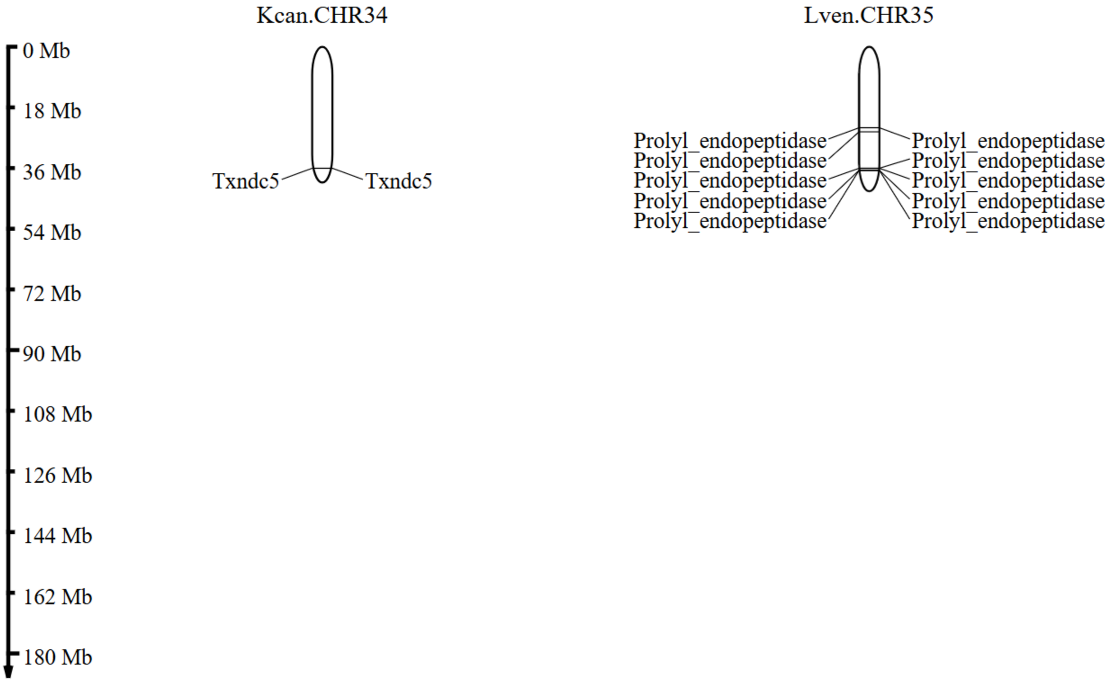
Kcan.CHR34
Lven.CHR35
0 Mb
18 Mb
36 Mb
54 Mb
72 Mb
90 Mb
108 Mb
126 Mb
144 Mb
162 Mb
180 Mb
Txndc5
Txndc5
Prolyl_endopeptidase
Prolyl_endopeptidase
Prolyl_endopeptidase
Prolyl_endopeptidase
Prolyl_endopeptidase
Prolyl_endopeptidase
Prolyl_endopeptidase
Prolyl_endopeptidase
Prolyl_endopeptidase
Prolyl_endopeptidase

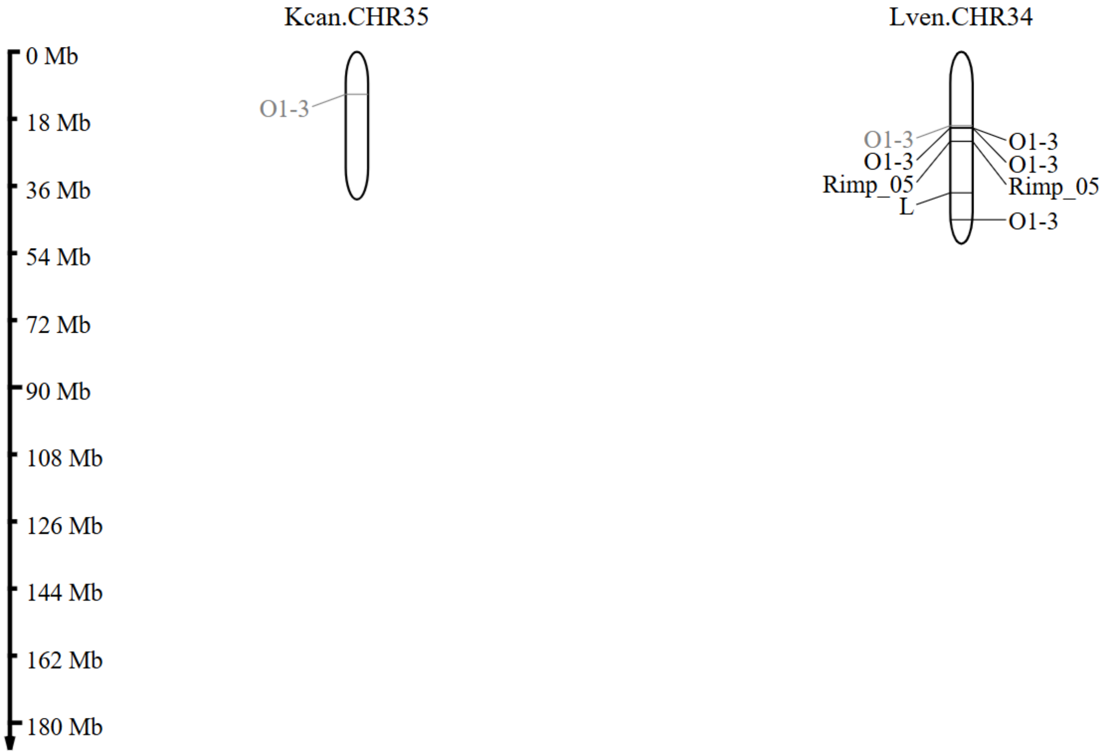
Kcan.CHR35
Lven.CHR34
0 Mb
18 Mb
36 Mb
54 Mb
72 Mb
90 Mb
108 Mb
126 Mb
144 Mb
162 Mb
180 Mb
O1-3
O1-3
O1-3
Rimp_05
L
O1-3
O1-3
Rimp_05
O1-3

Supplementary Figure S3. (A) Alignment of M3-a superfamily genes from OG001, with seven paralogs from *K. canariensis* (GENK_015-019, GENK_024, and GENK_029; and four paralogs from *L. ventricosus* (GENM_057- 060); (B) Alignment of Conkunitzin-12 precursor gene GENK070 from *K. canariensis* (two isoforms, t1 and t2) with GENM152 from *L. ventricosus* (OG077); (C) Alignment of A2 superfamily gene GENK136 from *K. canariensis* with *L. ventricosus* paralogs (GENM327, GENM328, and GENM329). The signal, propeptide, and mature regions (with their cysteine patterns) are shown.

A

```
[Superfamily M-3a]
[Framework III]
[                        /-------Signal--------/-----------------Pro-------------------/ /CC----C-----C-CC-----]

GENK_015                 ---MLKMGVVLFTFLVLFPLATLQLDA--DQPVERYVENKQDLNPDERRDFMVPIV-----RG  CCT--ACR-MPPCTCCW---
GENM__060                ---MLKMGVVLFTFLVLFPLATLQLDA------------------------------------  --------------------
TF39_113                 --------------------------------VERYVENKQDLNPDERRDFMVPIV-----RG  CCT--ACR-MPPCTCCW---
GENK_016                 ---MLKMGVVLFTFLVLFPLATFQLDA--DQPVERYAENKQDLNPDERRDFMVPIV-----RG  CCTA----------CRMP--
TF36_013                 ------MGVVLFTFLVLFPLATLQLDA--DQPVERYAENKQDLNPDERRDFMVPIV-----RG  CCTA----------CRMP--
GENK_017                 ---MLKMGVVLFTFLVLFPLATFQLDA--DQPIERYAENKQDLNPDERMEFILHAL----RRR  CCVSPAC--HDDCICCITR-
CV8_073                  --MMLNMGVVLFTFLVLFPLATLQLDA--DQPVERYVENKQDLNPDERMDFILHALG---QRR  CCSWYNC--WEDCVCCMPP-
TF39_114                 ------MGVVLFIFLVLFPLATLQLDA--DQPVERYVENKQDLNPDERMEFILHALG---QRR  CCVSPAC--HDDCICCITR-
GENK_019                 ------MGVVLFIFLVLFPLATLQLDA--DQPVERYAENKQDLNPDERMEFILHALG---QRR  CCVSPAC--HDDCICCITR-
GENK_029                 ---MLKMGVVLFTFLVLFPLATLQLDAGEDQTVERYAENKQDLNPDERREIILNVL-----GR  CCIPPMC--RSACSCCEKP-
GENM__058                --MMLNMGVVLFTFLVLFPLATLQMDA------ERYVENKQDLNPDERMDFIF-------QRR  CCSWYNC--WEDCVCCMPRA
GENM__057                --MMLKMGVMLFTFLVLFPLATLQLDT--DQPVERYVENKQDLNPDERMEIVWPALA---QVN  CCSLSACKPWSGVCCCA---
CV8_076                  --MMLKMAVMLFTFLVLFPLATLQLDA--DQPVERYAENTQDLNPDERREIISHALR---QVN  CCSLSACKPWSGCKCCA---
CV8_077                  ---MLKMRVVLFTFLVLFPLAMLQLDA--DQPRERYAENKQDLNQNERRDFVLHVL----RER  CCLGPTC--LEECYCCV---
GENM__059                ---MLKMRVVLFTFLVLFPLAMLQLDA--DQPRERYAENKQDLNQNERRDFVLHVL----RER  CCLGPTC--LEKCYCCV---

[Superfamily M-3a WF]
[No Framework]

GENK_024                 ---MLKMGVVLFIFLVLFPLATLQLDA--DQPVERYAENKQDLNPDERREIILPAL-------  --------------------
GENK_018                 MFVMLKMAVVLFTFLVLFPLATLQLDA--DQPVERYAENKQDLNPDERMEIILSAL----R--  --------------------
TF42_060                 ------------------PLATLQLDA--DQPVERYAENKQDLNPDERREIILPALGHGRSSR  CSGWRTC-------------
```

B

```
Conkunitzin-12
[Framework XXII]
[                            /--------Signal------/ /--C------------------------C--------------------C--C---C-C------------------------C--------------------C---C--]
                                                      /----------------------KU--motif----------------------/ /---------------------KU--motif-------------------/
TF42_027                     MDALRFSAVLLILAMACFMTES TESCDKAKDEGTGNQNIASYFFDSATGTCETFFYKGSGNNDPFPNRFNSHAE----CKCKLRMDVGTGRRRRTRYYHNTDGSGCLPFQYTGTGGNTNNFAYKWDCQNECE-
GENK070t2  MLDCTAQQQRSLDSSERSMDALRFSAVLLILAMACFMTES TESCDKAKDEGTGNQNIASYFFDSATGTCETFFYKGSGNNDPFPNRFNSHAE----CKCKLRMDVGTGRRRRTRYYHNTDGSGCLPFQYTGTGGNTNNFAYKWDCQNECE-*
GENK070t1  MLDCTAQQQRSLDSSERSMDALRFSAVLLILAMACFMTES TESCDKAKDEGTGNQNIASYFFDSATGTCETFFYKGSGNNDPFPNRFNSHAECENICKCKLRMDVGTGRRRRTRYYHNTDGSGCLPFQYTGTGGNTNNFAYKWDCQNECE-*
GENM_152                      MALRFSAVLLILAMACVMIES T-VCDKAKVEGTGSLNLTRHFFNNDTGTCETFYYTG-GNDDTFPNRFNFNSVCENTCQVQTRKDPGTGQRVTTRYYHSSDGSGCQSFQYRGNGGNANNFDEKSECINECE-*
CV8_028                      MDALRFSAVLLILAMACVMIES TAVCDKAKVEGTGSLNLTRHFFNNDTGTCETFYYTG-GNDDTFPNRFNFNSVCENTCKCYLKMDQGTGQSPRTRYYHNTDGSGCQSFQYQGSGGNANNFDSESACENECES
```

C

```
A2 Superfamily alpha 4/7
[Framework I]
[                                 /------Signal--------/ /--------Pro--------/ /-----Mature------]
                                                                               /-CC----C-------C-]
A_1387_008                        MRCLAFLVVTLLLFTAMETTGA SNRVNAAANGKTSDSISLAVR DDCCPNPSCRQNHPELCA
GENK_136                          MRCLAFLVVTLLLFTAMATTG- GNRVNAAASGKASDSISLAVR DDCCPSPSCRQNHPELCS*
GENM_328                          MRCLAFLVVTLLLFTAMATTG- SNRVNAAANGKASASISLAVR DDCCPNPSCRQNHPELCR*
CV10_004                          ---------------------A STRVNAAASGKASDSISLAVR DGCCSNPACHQNHPEICR
GENM_327                          MRCLAFLVVTLLLFTAMATTG- --------------------- ------------------
GENM_329                          MRCLAFLVVTLLLFTAMATTG- --------------------- ------------------
```
