# Supplementary material for: Comparative analysis of venom genes in the chromosome-level genomes of two closely related cone snails
Source: BMC Genomics. 2026 Mar 18;27:409. doi: 10.1186/s12864-026-12745-4 (PMC13112664; doi:10.1186/s12864-026-12745-4)
Supplement: Supplementary file 1 — Supplementary Material 1. [file 12864_2026_12745_MOESM1_ESM.zip › SupplMattNew/Supplementary Material Captions New.docx]

**Description of Supplementary material**

Additional file 1**- SupplementaryFigures.pdf**

- Title: Supplementary Figures

-Description: Collation of all supplementary figures referred to in the main text

Additional file 2**- Supp File S1.fasta**

**-** Title: Supplementary File S1**.**

**-** Description: Different conotoxin precursor, hormones and venom-related sequences from venom gland transcriptomes (TF39, TF42, and TF43) of *K. canariensis* in a FASTA file.

Additional file 3 **- Supp File S2.txt**

**-** Title: Supplementary File S2.

**-** Description: Alignments of conotoxin precursors, hormones, and associated venom proteins of *K. canariensis* (TF39, TF42, and TF43) with homologues from other cone snail species.

Additional file 4 - **Supp File S3.gff3**

Title: Supplementary File S3.

- Description: Venom gene annotations of *K. canariensis* in GFF3 format.

Additional file 5 **– Supp File S4.fasta**

-Title: Supplementary File S4.

Description: Nucleotide sequences (CDS) of annotated venom genes from the *K. canariensis* genome in FASTA format.

Additional file 6 **– Supp File S5.fasta**

**-**Title: Supplementary File S5.

-Description: Amino acid sequences of annotated venom genes from the *K. canariensis* genome in FASTA format. The conotoxin gene superfamily, as well as the hormone and venom-related gene family, is indicated in the sequence headers, following a double underscore.

Additional file 7 **– Supp File S6.txt**

- Title: Supplementary File S6.

**-**Description: Venom-related genes in the *K. canariensis* genome. Annotation of conotoxin precursors, hormones, and other venom-related protein genes ordered by their location in the 35 pseudochromosomes. The nucleotide sequence of the gene (partitioned in exons) and the amino acid sequence of the derived protein are given. Coordinates of the gene sequence are provided (the sense of the arrow indicates forward or reverse transcription).

Additional file 8 **– Supp Table S1.xlsx**

Titles: Supplementary Table S1.

- Description: Sequencing and assembly statistics of the three specimens of *K. canariensis* analyzed in this work.

Additional file 9 **– Supp Table S2.xlsx**

Titles: Supplementary Table S2.

- Description: Transcriptomes of the venom glands of *K. canariensis* (**worksheets 1-4**: TF39, TF42, TF43 and common conotoxin precursor transcripts) and comparative summary of conotoxin precursor superfamilies between *K. canariensis* (Kcan; TF39) and *L. ventricosus* (Lven; CV8) (**worksheet 5**).

Additional file 10 **– Supp Table S3.xlsx**

**-**Title: Supplementary Table S3**.**

**-** Description: Venom (conotoxin precursor, hormone, venom-related) genes in the 35 pseudochromosomes of the *K. canariensis* genome assembly.

Additional file 11 **– Supp Table S4.xlsx**

- Title: Supplementary Table S4.

- Description: Number of conotoxin precursor genes in *K. canariensis* and *L. ventricosus*. Gene counts by conotoxin superfamily (**worksheet 1**) and by pseudochromosome (**worksheet 2**). Detailed counts by subfamily (**worksheet 3**).

Additional file 12 **– Supp Table S5.xlsx**

- Title: Supplementary Table S5.

- Description: Orthogroup inference between *K. canariensis* and *L. ventricosus*. Venom genes within orthogoups between *K. canariensis* (GENK) and *L. ventricosus* (GENM), including syntenic regions (+-200000 pb) and their percent identity, aligned length, and genomic coordinates in both species. Gene superfamilies are indicated along with their chromosomal locations. Abbreviations: **CTX**, conotoxin; **OG**, orthogroup; **HChr**, homologous pseudochromosome; **NHChr**, non-homologous pseudochromosome (**worksheet 1**); Orthogrups assigned with orthofinder, including the number of venom genes identified per species with their chromosomal location and orthogroup (**worksheet 2**).

Additional file 13 **– Supp Table S6.xlsx**

- Title: Supplementary Table S6.

- Description: Comparative analysis of venom gene regions between K. canariensis and L. ventricosus. (**worksheet 1**) Summary of sequence alignment metrics for flanking regions between both species. (**worksheets 2 and 3**) Genomic regions (±200 kb) containing venom genes that are present in one species but absent in the other, showing mean percent identity and aligned length percentage. Regions with > 80% of sequence identity in > 50% of the alignment are highlighted in bold, within this regions, those located on non-homologous pseudochromosomes are shaded in yellow.

Additional file 14 **– Supp Table S7.xlsx**

- Title: Supplementary Supplementary Table S7.

- Description: Summary of venom gland transcriptomes reported from different cone snail species. Number of conotoxin precursor transcripts and gene superfamilies identified in each species based on published transcriptomic datasets and this study.
